# A Chaotic Electromagnetic Field Optimization Algorithm Based on Fuzzy Entropy for Multilevel Thresholding Color Image Segmentation

**DOI:** 10.3390/e21040398

**Published:** 2019-04-15

**Authors:** Suhang Song, Heming Jia, Jun Ma

**Affiliations:** College of Mechanical and Electrical Engineering, Northeast Forestry University, Harbin 150040, China

**Keywords:** fuzzy entropy, electromagnetic field optimization, chaotic strategy, color image segmentation, multilevel thresholding

## Abstract

Multilevel thresholding segmentation of color images is an important technology in various applications which has received more attention in recent years. The process of determining the optimal threshold values in the case of traditional methods is time-consuming. In order to mitigate the above problem, meta-heuristic algorithms have been employed in this field for searching the optima during the past few years. In this paper, an effective technique of Electromagnetic Field Optimization (EFO) algorithm based on a fuzzy entropy criterion is proposed, and in addition, a novel chaotic strategy is embedded into EFO to develop a new algorithm named CEFO. To evaluate the robustness of the proposed algorithm, other competitive algorithms such as Artificial Bee Colony (ABC), Bat Algorithm (BA), Wind Driven Optimization (WDO), and Bird Swarm Algorithm (BSA) are compared using fuzzy entropy as the fitness function. Furthermore, the proposed segmentation method is also compared with the most widely used approaches of Otsu’s variance and Kapur’s entropy to verify its segmentation accuracy and efficiency. Experiments are conducted on ten Berkeley benchmark images and the simulation results are presented in terms of peak signal to noise ratio (PSNR), mean structural similarity (MSSIM), feature similarity (FSIM), and computational time (CPU Time) at different threshold levels of 4, 6, 8, and 10 for each test image. A series of experiments can significantly demonstrate the superior performance of the proposed technique, which can deal with multilevel thresholding color image segmentation excellently.

## 1. Introduction

Image segmentation is an important technology in image processing, which is a frontier research direction in computer vision, as well as one of the key preprocessing steps in image analysis [1,2]. It has been widely adopted in medicine, agriculture, industrial production, and various other fields. Image segmentation can be defined as the procedure of dividing an image into different regions [3]. In the subsequent research, the relevant regions can be extracted from the segmented image expediently according to specific requirements. Nowadays, the common image segmentation methods include threshold-based, cluster-based, edge-based methods and so on. Thresholding is extensively applied due to its simplicity, efficiency, and robustness. Depending on the number of thresholds, it can be classified as bi-level segmentation and multilevel segmentation [4]. Bi-level thresholding techniques use one threshold to partition an image into two segments; whereas multilevel segmentation determines several thresholds to separate an image into more than two classes. Many thresholding approaches have been proposed by scholars around the world in the past few years, Otsu’s (between-class variance criterion) [5,6] technique pushes the thresholding segmentation to an upsurge and inspires the scholars constantly in this field. Then diverse entropy-based criteria have emerged in the thresholding segmentation study, such as maximum entropy (Kapur’s) [7], minimum cross entropy [8], fuzzy entropy [9], etc. 

Gray-scale image thresholding technology is relatively popular and mature. Compared with the segmentation of gray-scale images, color image segmentation plays a more beneficial role in practical applications, which separates an image into several disjoint and homogenous components based on the information of texture, color or histogram [10]. Color image segmentation is more complex and challenging than gray-scale images. Nevertheless, considering that color images contain more characteristics and they are closer to human visual effects [11], the research of color image segmentation is more meaningful. There will appear some problems when a traditional segmentation method is adopted to segment a color image, for example, the computation is massive and accuracy of segmented images cannot be guaranteed [12,13]. In this paper, fuzzy entropy is one of the research objects with high segmentation accuracy. In the fuzzy entropy thresholding technique, each threshold needs to be determined by three fuzzy parameters. Hence the calculation of thresholds is more accurate, at the same time the process is more complicated and the running time of the program will be longer. With the improvement of the threshold level, the computation of fuzzy entropy will exponentially increase for searching the optimal thresholds and the efficiency of segmentation will gradually decrease [14,15,16]. In order to enhance the practicability of fuzzy entropy thresholding technique, this paper combines fuzzy entropy thresholding with intelligent optimization algorithms to improve the performance with respect to accuracy and efficiency. 

Meta-heuristic algorithms are utilized to obtain the optimal solution of the problem [17]. Generally, they are inspired by nature and try to handle the problems from mimicking ethology, biology or physics [18]. For instance, Bird Swarm Algorithm (BSA) [19], Firefly Algorithm (FA) [20], and Flower Pollination Algorithm (FPA) [21] are inspired by ethology or biology; Electromagnetic Optimization (EMO) [22], Wind Driven Optimization (WDO) [23], and Gravitational Search Algorithm (GSA) [24] are inspired by physics. At present, a number of scholars have coupled the optimization algorithms with the field of image segmentation in the literature. For instance, Sowjanya et al. [25] combined a WDO algorithm with Otsu’s method for the segmentation of brain MRI images, it has shown the superior performance in the experiment results. Wasim et al. [26] proposed an improved Bee Algorithm (BA) for multilevel image segmentation, whereby they embedded Levy fight into a Bees Algorithm (the Levy Bees Algorithm, LBA), and the results show that LBA is more stable than BA in this field. Rakoth et al. [27] tried to combine Dragonfly Optimization with Self-Adaptive weight (SADFO) and used SADFO for image segmentation experiments with satisfying results. These references confirm the feasibility of applying optimization algorithms to image thresholding segmentation. However, the above experiments all concentrate on gray-scale images and do not extend the experiments to the analysis of color images. Applying meta-heuristic algorithms to the field of multilevel image segmentation can enhance the convergence speed and efficiency [28]. Therefore, in this paper, Electromagnetic Field Optimization algorithm (EFO) [29] is modified and combined with fuzzy entropy thresholding method to eliminate the complex computation, which is used into the multilevel color image segmentation field for searching the best threshold values. 

Electromagnetic Field Optimization is a new meta-heuristic algorithm inspired by the electromagnetic theory developed in physics. EFO algorithm has been applied in several applications, for example, Behnam et al. [30] created a method using EFO for hiding sensitive rules simultaneously, which has fewer lost rules than other well-known algorithms. Bouchekara et al. [31] proposed the optimal coordination of directional overcurrent relays based on EFO, and the results show that EFO is better than other optimization algorithms such as Particle Swarm Optimization (PSO) [32], or the Differential Evolution (DE) algorithm [33], etc. This paper embeds a new chaos strategy into standard EFO algorithm according to the specific problem of color image segmentation named as Chaotic Electromagnetic Field Optimization (CEFO). Employing the CEFO algorithm to optimize the fuzzy parameters which determine the optimal thresholds of an image in fuzzy entropy. To the best of our knowledge, this topic has not been investigated yet. The rest of this paper is organized as follows: in Section 2, the concept of EFO algorithm is elaborated. In Section 3, the chaotic strategy in CEFO algorithm is introduced and explained. In Section 4, the problem definitions and formulas of the Otsu’s, Kapur’s entropy, and the fuzzy entropy are illustrated. In Section 5, the experimental environment is reported. In Section 6, the experimental results and discussions are provided and analyzed. Finally, a brief conclusion of this paper and future works are drawn in Section 7.

## 2. Electromagnetic Field Optimization

Electromagnetic Field Optimization is a novel meta-heuristic intelligent algorithm proposed by Hosein in 2016 [29]. In contrast to the swarm-based meta-heuristic algorithms widely inspired by biology, the EFO algorithm is based on the electromagnetic field principle used in physics. In the EFO algorithm, due to the forces of attraction and repulsion in the electromagnetic field, the electromagnetic particle (EMP) keeps away from the worst solution and moves towards the best solution. In the end, all the electromagnetic particles (EMPs) gather around the optimal solution. 

A magnetic field is generated around the electrified iron core, which is made of an electromagnet. An electromagnet has only one polarity and it is contingent on the direction of the electric current. Hence, an electromagnet has two characteristics of attraction or repulsion, electromagnets with the different polarity attract each other, and those with identical polarity repel each other. The intensity of attraction is 5-10% higher than repulsion and the ratio between attraction and repulsion is set as golden ratio [29,31], which can promote the algorithm to explore the optimal solution effectively in the search space. The essence of the optimization problem is to find the pole (maximum or minimum) about the objective function and the corresponding fitness in the prescriptive range [34]. Each potential solution of the problem is represented with an electromagnetic particle composed of a group of electromagnets. The electromagnetic field comprises several electromagnetic particles and it can be defined as a space in 1-D (dimension), 2-D, 3-D, or hyperdimensional space [35]. The number of electromagnets of an electromagnetic particle corresponds to variables of the optimization problem, as well as the dimension of the electromagnetic space. Moreover, all electromagnets of one electromagnetic particle have the same polarity. Therefore, an electromagnetic particle has the same polarity with its electromagnets. The set of electromagnetic particles can be considered in a matrix as:

(1) $$EMPs=\left[\begin{array}{cccc} P_{1,1} & P_{1,2} & \cdots & P_{1,d}\\ P_{2,1} & P_{2,2} & \cdots & P_{2,d}\\ \vdots & \vdots & \vdots & \vdots \\ P_{n,1} & P_{n,2} & \cdots & P_{n,d} \end{array}\right]$$

where n is the number of electromagnetic particles and j is the number of variables (dimension).

The mechanism of the EFO algorithm can be described as follows:

*Step 1*: A certain number of electromagnetic particles are generated randomly in the electromagnetic field, and the fitness of each electromagnetic particle is evaluated by the objective function. Then the electromagnetic particles are sorted on the basis of their fitness.
*Step 2*: The electromagnetic field is divided into three regions: positive, negative and neutral. Then all electromagnetic particles are classified into these three groups. The first group consists of the best particles with positive polarity. The second group consists of the worst particles with negative polarity. The third group consists of neutral particles which have a little negative polarity almost near zero. And all electromagnetic particles are located in the corresponding electromagnetic regions.
*Step 3*: In each iteration of the algorithm, a new electromagnetic particle ($EMP^{New}$) is generated. If the fitness of $EMP^{New}$ is better than the original worst particle, the $EMP^{New}$ will remain and its fitness and polarity will depend on the list of fitness, furthermore, the original worst particle will be eliminated. If else, the will be eliminated directly. This process continues until the algorithm reaches the maximum number of iterations.

**Figure 1:**
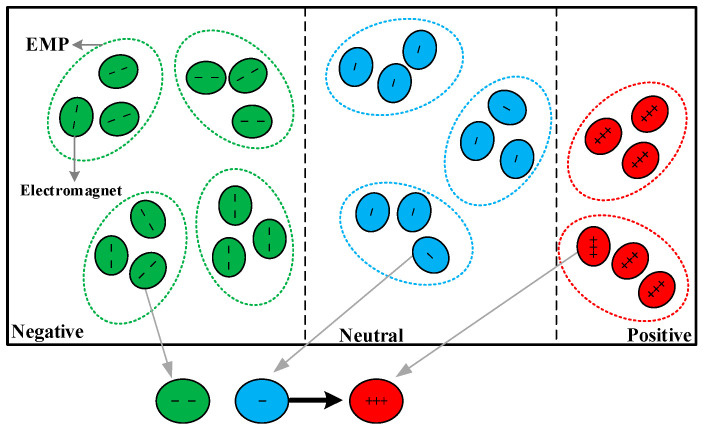
Schematic diagram of the electromagnetic field. The relationship between the electromagnetic particle (EMP) and electromagnet. The method of generating the new electromagnetic particle

The core of the EFO is the method of generating $EMP^{New}$ in each iteration, and each electromagnet in $EMP^{New}$ is shaped separately. The main process can be described as follows: three electromagnetic particles are randomly extracted from three electromagnetic regions (one EMP from each region), and then three electromagnets are randomly extracted from three electromagnetic particles obtained just now (one electromagnet from each EMP). Consequently, there are three electromagnets with different polarities. The neutral electromagnet is attracted and repelled by positive and negative electromagnets. Owing to the intensity of attraction is stronger than repulsion and the neutral electromagnet has a slight negative polarity, the neutral electromagnet moves a distance away from the negative electromagnet and approaches towards the positive electromagnet. In other words, each electromagnet in $EMP^{New}$ is a result of interaction between attraction and repulsion, which is shown in Figure 1. 

Figure 1 shows the process of generating $EMP^{New}$, in this figure, each electromagnetic particle contains three electromagnets for example, and positive, neutral and negative electromagnets are colored as green, blue and red respectively. In accordance with the above mechanism, three electromagnets of $EMP^{New}$ is selected from nine original electromagnets, which increases randomness and enhances the strength of the optimization algorithm. Establishing a mathematical model to describe the update mechanism of $EMP^{New}$ as below:

(2) $$D_{j}^{P_{j}K_{j}}=EMP_{j}^{P_{j}}-EMP_{j}^{K_{j}}$$

(3) $$D_{j}^{N_{j}K_{j}}=EMP_{j}^{N_{j}}-EMP_{j}^{K_{j}}$$

(4) $$EMP_{j}^{New}=EMP_{j}^{K_{j}}+[(\varphi *r)*D_{j}^{P_{j}K_{j}}]-(r*D_{j}^{N_{j}K_{j}})$$

where *j* is the number of electromagnets in EMP; $EMP_{j}^{P_{j}}$ is the positive electromagnet; $EMP_{j}^{N_{j}}$ is the negative electromagnet; $EMP_{j}^{K_{j}}$ is the neutral electromagnet; $D_{j}^{P_{j}K_{j}}$ is the distance between positive and neutral electromagnets. $D_{j}^{N_{j}K_{j}}$ is the distance between negative and neutral electromagnets; r is the random value between 0 and 1; φ is the golden ratio of $(\sqrt{5}+1)/2$. 

In order to preserve the diversity of particles in the electromagnetic field and reduce the probability of falling into local optima [36], randomness is an indispensable part in EFO algorithm. Therefore, the probability of Ps_rate about the new position is determined by the selected electromagnet from a positive field, which accelerates the convergence rate and improves the accuracy of the optimum. Additionally, the probability of R_rate is used to replace one electromagnet in $EMP^{New}$ with randomly generated electromagnet within the space. The most important feature of EFO algorithm is the high degree of cooperation among particles. Another pivotal characteristic is high randomization, which avoids obtaining the local optimum. Meanwhile, the application of the golden ratio makes EFO more efficient. All of the above strategies lead EFO to a robust optimization algorithm. 

## 3. Proposed Algorithm

One of the essential points in the EFO algorithm is the degree of chaos about the electromagnetic particles in the electromagnetic field; if the degree of chaos is higher, the search power will be stronger. In the literature, the initial position of electromagnetic particles is processed by a chaotic strategy, which disturbs the distribution of particles and increases the unpredictability of the system. 

Chaotic phenomena refer to the external complex behavior in a non-linear deterministic system due to the inherent randomness [37]. Almost all meta-heuristic algorithms need to be initialized randomly, and usually it is achieved by using probability distribution, which can advantageous to replace such randomness with chaotic map [38]. Owing to the dynamic behavior of chaos, chaotic maps have been commonly acknowledged in the field of optimization, which can promote algorithms in exploring optima more effective globally in the search space. Table 1 lists some common chaotic maps, which are expressed by mathematical equations. 

**Figure 2:**
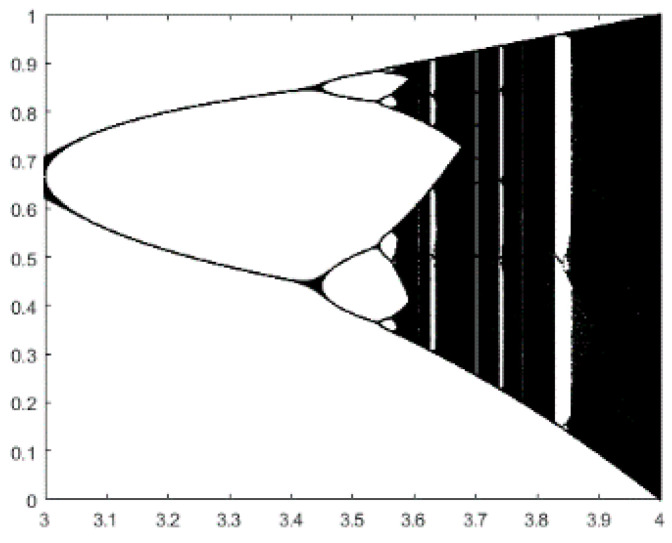
Logistic Chaotic Map.

For instance, logistic chaos is widely used because of its simple expression and good performance, and it is shown in Figure 2. As can be seen, the logistic system has missed certain values. In consideration of the multilevel color image segmentation problem, this paper proposes a new chaotic map as follows:

(5) $$x_{n+1}=rand()\times \sin(2\pi x_{n})+x_{n}$$

where rand() is the random value between 0 and 1.

**Figure 3:**
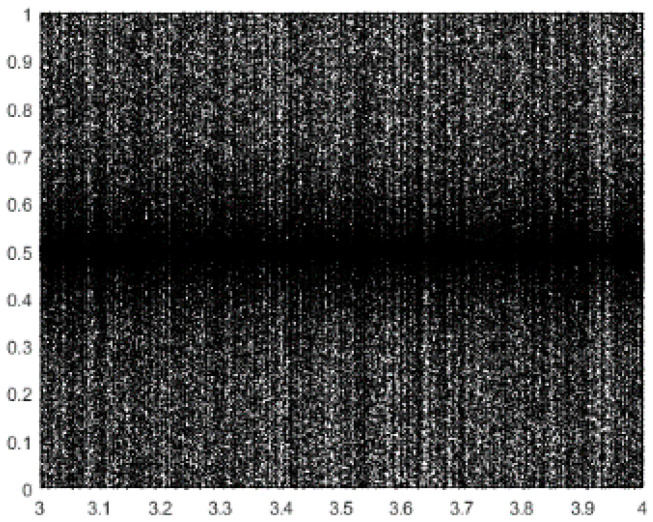
Chaotic Map in this paper.

The new chaotic map is shown in Figure 3 and its distribution is more symmetrical than Logistic chaotic map. Taking advantage of this chaos strategy in EFO, the total performance of the algorithm will be improved and it is known as CEFO. The pseudo-code of the CEFO algorithm is presented in Algorithm 1. 

**Algorithm 1.** Pseudo-code of CEFO algorithm
/* Part 1: Algorithm parameters initialization */
 N_var: The number of electromagnets in each electromagnetic particle. N_emp: The number of electromagnetic particles in population. Ps_rate: The probability of changing one electromagnet with a random electromagnet. R_rate: The probability of selecting electromagnets from the positive field. P_field: The portion of particles belonging to positive. N_field: The portion of particles belonging to negative. min = lower boundary; max = upper boundary
/* Part 2: Main loop of the algorithm */
 **for**
*i* = 1 to N_emp
**do**   **for**
*j* = 1 to N_var
**do**    position [*i*, *j*] = min + rand ( )∗(max − min)   **end for** **end for** **Update** position by using the chaotic map of Equation (5) fitness = function (position) **while** t (current iteration) < max iterations   Divide the electromagnetic field into three regions   **for**
*i* = 1 to N_var
**do**     **if** rand (0,1) > Ps_rate
      Generate the $EMP^{New}$ by Equation (4)     **else**
      Generate the $EMP^{New}$ from positive particles     **end if**     Check if any particle beyond the search space   **end for**   **if** rand (0,1) < R_rate
     Change one electromagnet of $EMP^{New}$ randomly   **end if**
   Compare the fitness of $EMP^{New}$ with worst particle   *t* = *t* + 1 **end while** Output the best particle

## 4. Thresholding Segmentation Methods

The process of multilevel thresholding color image segmentation is to find more than two optimal thresholds to segment three components (red, green, and blue) respectively. In RGB images, each color component consists of P pixels and L number of gray levels. The obtained thresholds are within the range of [0, L−1], L is considered as 256 and each gray-level is associated with the histogram representing the frequency of its gray level pixel used by g(x,y). 

### 4.1. Between-Class Variance Thresholding

Between-class variance (Otsu’s) [5] thresholding method can be defined as follows: 

Assuming that n−1 thresholds form the threshold vector $T=\left[t_{1},t_{2},\cdots ,t_{n-1}\right]$ to split an image into *n* classes:

(6) $$\left\{ \begin{array}{l} C_{1}=\left\{(x,y)|0\leq g(x,y)\leq t_{1}-1\right\}\\ C_{2}=\left\{(x,y)|t_{1}\leq g(x,y)\leq t_{2}-1\right\}\\ \vdots \\ C_{n}=\left\{(x,y)|t_{n-1}\leq g(x,y)\leq L-1\right\} \end{array}\right.$$

Constructing image histogram $\left\{f_{0},f_{1},\cdots ,f_{L-1}\right\}$, where $f_{i}$ is the frequency of gray-level i. Then, the probability of gray-level i can be represented as:

(7) $$p_{i}=\frac{f_{i}}{\sum_{i=0}^{L-1}f_{i}},\;\sum_{i=0}^{L-1}p_{i}=1$$

For every class $C_{k}$, the cumulative probability $\omega_{k}$ and average gray level $\mu_{k}$ in every region can be defined as:

(8) $$\omega_{k}=\sum_{i\in C_{k}}p_{i},\;\mu_{k}=\sum_{i\in C_{k}}\frac{i\cdot p_{i}}{\omega_{k}}$$

and Otsu’s function can be expressed as:

(9) $$\sigma_{B}^{2}=\sum_{k=0}^{K}\omega_{k}\cdot {\left(\mu_{k}-\mu_{T}\right)}^{2},\;\mu_{T}=\sum_{i=0}^{L-1}i\cdot p_{i}$$

where $\mu_{k}$ is the average gray intensity of the image.

Therefore, the optimal threshold vector is as follows:

(10) $$T^{*}=\arg\max(\sigma_{B}^{2})$$

### 4.2. Kapur’s Entropy Thresholding

Kapur’s entropy method maximizes the entropy value of the segmented histogram such that each separated region has more centralized distribution [39]. Extending Kapur’s entropy for multilevel image segmentation problem:

(11) $$\begin{array}{l} H_{1}=-\sum_{i=0}^{t_{1}-1}\frac{p_{i}}{\omega_{1}}\ln\frac{p_{i}}{\omega_{1}},\;\;\omega_{1}=\sum_{i=0}^{t_{1}-1}p_{i}\\ H_{2}=-\sum_{i=t_{1}}^{t_{2}-1}\frac{p_{i}}{\omega_{2}}\ln\frac{p_{i}}{\omega_{2}},\;\;\omega_{2}=\sum_{i=t_{1}}^{t_{2}-1}p_{i}\\ H_{j}=-\sum_{i=t_{j-1}}^{t_{j}-1}\frac{p_{i}}{\omega_{j}}\ln\frac{p_{i}}{\omega_{j}},\;\;\omega_{j}=\sum_{i=t_{j-1}}^{t_{j}-1}p_{i}\\ H_{n}=-\sum_{i=t_{n}}^{L-1}\frac{p_{i}}{\omega_{n}}\ln\frac{p_{i}}{\omega_{n}},\;\;\omega_{n}=\sum_{i=t_{n}}^{L-1}p_{i} \end{array}$$

where $H_{j}$ represents the entropy value of *j*-th region in the image. 

There are n thresholds which can be configured as the n dimensional optimization problem. And the optimal threshold vector is obtained analogously by:

(12) $$T^{*}=\arg\max(\sum_{i=0}^{m}H_{i})$$

### 4.3. Fuzzy Entropy Thresholding

In the fuzzy entropy technique, let an original image be D={(i,j)|i=0,⋯,M−1;j=0,⋯,N−1}, where M and N represent the width and height of an image. Supposed that $t_{1}$ and $t_{2}$ are two thresholds to divide the original image into 3 parts named as $E_{d}$, $E_{m}$, $E_{b}$ [10]. $E_{d}$ consists of pixels of low gray levels; $E_{m}$ is made of pixels with middle gray levels; $E_{b}$ is composed of pixels of high gray levels. Usually, using (13) to calculate the image histogram:

(13) $$h_{k}=\frac{n_{k}}{M*N}$$

where k=0,1,⋯,255; $n_{k}$ is the number of the *k*-th pixel in $D_{k}$; $h_{k}$ is the histogram of the image at gray-level k, $\sum_{k=0}^{255}h_{k}=1$.

Consider $\Pi_{3}=\left\{E_{d},E_{m},E_{b}\right\}$ as an unknown probabilistic partition of D, whose probability distribution can be expressed as:

(14) $$p_{d}=P(E_{d});\;\;\;\;\;\;p_{m}=P(E_{m});\;\;\;\;\;p_{b}=P(E_{b})$$

For each (i,j)∈D, let:

(15) $$\begin{array}{l} D_{d}=\{(i,j)|0\leq g(i,j)\leq t_{1}\},\\ D_{m}=\{(i,j)|t_{1}\leq g(i,j)\leq t_{2}\},\\ D_{b}=\{(i,j)|t_{2}\leq g(i,j)\leq L-1\} \end{array}$$

**Figure 4:**
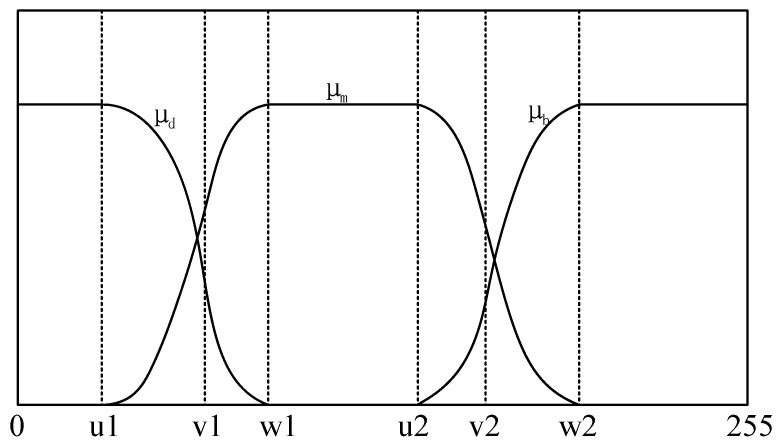
Membership function graph.

Utilizing $\mu_{d}$, $\mu_{m}$, $\mu_{b}$ as the membership functions of $E_{d}$, $E_{m}^{}$, $E_{b}$, which is shown in Figure 4 [40]. There are six fuzzy parameters of $u_{1}$, $v_{1}$, $w_{1}$, $u_{2}$, $v_{2}$, $w_{2}$ in the membership functions, in other words, $t_{1}$ and $t_{2}$ are determined by these six parameters. According to the above statement, we can have the probability distribution of three regions expressed as:

(16) $$\begin{array}{l} p_{d}=\sum_{k=0}^{255}p_{k}\cdot p_{d|k}=\sum_{k=0}^{255}p_{k}\cdot \mu_{d}(k)\\ p_{m}=\sum_{k=0}^{255}p_{k}\cdot p_{m|k}=\sum_{k=0}^{255}p_{k}\cdot \mu_{m}(k)\\ p_{b}=\sum_{k=0}^{255}p_{k}\cdot p_{b|k}=\sum_{k=0}^{255}p_{k}\cdot \mu_{b}(k) \end{array}$$

where $p_{d|k}$, $p_{m|k}$, $p_{b|k}$ are the conditional probability of a pixel partitioned into three classes. Moreover, a pixel of k in an image satisfies the constraint of $p_{d|k}+p_{m|k}+p_{b|k}=1$.

The three membership functions have been shown in Figure 4. And these mathematical formulas are defined as follows:

(17) $$\mu_{d}(k)=\left\{ \begin{array}{ll} 1 & k\leq u_{1}\\ 1-\frac{{\left(k-u_{1}\right)}^{2}}{\left(w_{1}-u_{1})\cdot (v_{1}-u_{1}\right)} & u_{1}\leq k\leq v_{1}\\ \frac{{\left(k-w_{1}\right)}^{2}}{\left(w_{1}-u_{1})\cdot (w_{1}-v_{1}\right)} & v_{1}\leq k\leq w_{1}\\ 0 & k\geq w_{1} \end{array}\right.$$

(18) $$\mu_{m}(k)=\left\{ \begin{array}{ll} 0 & k\leq u_{1}\\ \frac{{\left(k-u_{1}\right)}^{2}}{\left(w_{1}-u_{1})\cdot (v_{1}-u_{1}\right)} & u_{1}\leq k\leq v_{1}\\ 1-\frac{{\left(k-w_{1}\right)}^{2}}{\left(w_{1}-u_{1})\cdot (w_{1}-v_{1}\right)} & v_{1}\leq k\leq w_{1}\\ 1 & w_{1}\leq k\leq u_{2} \end{array}\right.$$

(19) $$\mu_{b}(k)=\left\{ \begin{array}{ll} 0 & k\leq u_{2}\\ \frac{{\left(k-u_{2}\right)}^{2}}{\left(w_{2}-u_{2})\cdot (v_{2}-u_{2}\right)} & u_{2}\leq k\leq v_{2}\\ 1-\frac{{\left(k-w_{2}\right)}^{2}}{\left(w_{2}-u_{2})\cdot (w_{21}-v_{2}\right)} & v_{2}\leq k\leq w_{2}\\ 1 & k\geq w_{2} \end{array}\right.$$

where $u_{1}$, $v_{1}$, $w_{1}$, $u_{2}$, $v_{2}$, $w_{2}$ should meet the condition of $0\leq u_{1}<v_{1}<w_{1}<u_{2}<v_{2}<w_{2}\leq 255$.

Then, the fuzzy entropy of each part is as follows:

(20) $$\begin{array}{l} H_{d}=-\sum_{k=0}^{255}\frac{p_{k}\cdot \mu_{d}(k)}{p_{d}}\cdot \ln(\frac{p_{k}\cdot \mu_{d}(k)}{p_{d}})\\ H_{m}=-\sum_{k=0}^{255}\frac{p_{k}\cdot \mu_{m}(k)}{p_{m}}\cdot \ln(\frac{p_{k}\cdot \mu_{m}(k)}{p_{m}})\\ H_{b}=-\sum_{k=0}^{255}\frac{p_{k}\cdot \mu_{b}(k)}{p_{b}}\cdot \ln(\frac{p_{k}\cdot \mu_{b}(k)}{p_{b}}) \end{array}$$

The whole fuzzy entropy function is defined as:

(21) $$H(u_{1},v_{1},w_{1},u_{2},v_{2},w_{2})=H_{d}+H_{m}+H_{b}$$

Equation (21) is determined by six variables which are called fuzzy parameters. Seeking the optimal group of $u_{1}$, $v_{1}$, $w_{1}$, $u_{2}$, $v_{2}$, $w_{2}$ when (21) reach the maximum value. Therefore, the most applicable threshold can be calculated as:

(22) $$\begin{array}{l} \mu_{d}(t_{1})=\mu_{m}(t_{1})=0.5\\ \mu_{m}(t_{2})=\mu_{b}(t_{2})=0.5 \end{array}$$

As is shown in Figure 4, according to the above equation, $t_{1}$ and $t_{2}$ can be defined by (17)–(19), and the result is as follows:

(23) $$\begin{array}{l} t_{1}=\left\{ \begin{array}{ll} u_{1}+\sqrt{(w_{1}-u_{1})\cdot (v_{1}-u_{1})/2} & (u_{1}+w_{1})/2<v_{1}<w_{1}\\ w_{1}-\sqrt{(w_{1}-u_{1})\cdot (w_{1}-v_{1})/2} & u_{1}<v_{1}<(u_{1}+w_{1})/2 \end{array}\right.\\ t_{2}=\left\{ \begin{array}{ll} u_{2}+\sqrt{(w_{2}-u_{2})\cdot (v_{2}-u_{2})/2} & (u_{2}+w_{2})/2<v_{2}<w_{2}\\ w_{2}-\sqrt{(w_{1}-u_{2})\cdot (w_{2}-v_{2})/2} & u_{2}<v_{2}<(u_{2}+w_{2})/2 \end{array}\right. \end{array}$$

Fuzzy entropy thresholding can meet the requirement from single threshold segmentation to multiple thresholds segmentation, and the optimal threshold vector obtained is more precise. However, each threshold should be determined by three parameters in fuzzy entropy thresholding, and thresholds need to be defined by 3*n* fuzzy parameters [41,42]. 

**Figure 5:**
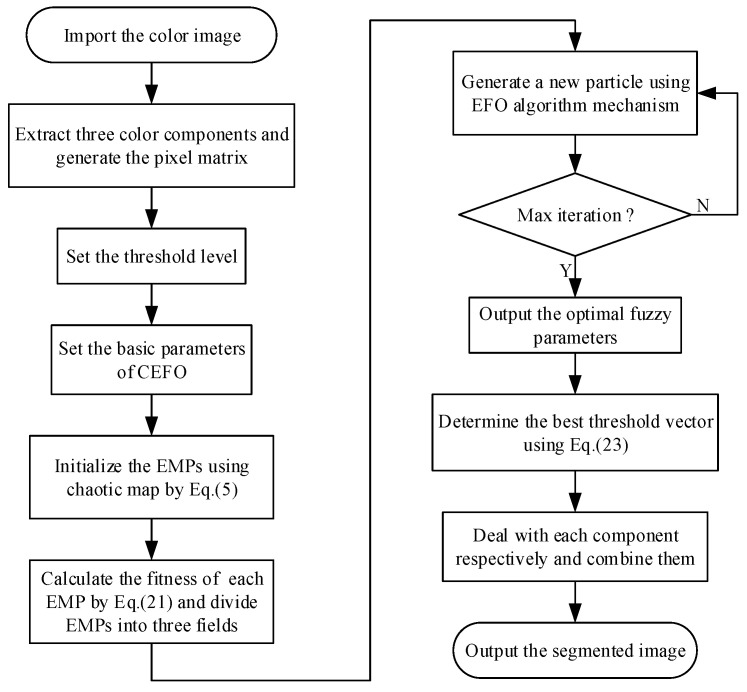
Flow chart of fuzzy entropy thresholding method based on the Chaotic Electromagnetic Field Optimization (CEFO) algorithm.

With the increase of threshold level gradually, the degree of the computation will be significantly risen, which diminish the speed of the process and the practicability will be reduced. In order to improve the convergence efficiency, it is necessary to use the optimization algorithm for searching the optimal threshold vector. This paper takes advantage of the CEFO to ensure the segmentation accuracy and greatly decrease the execution time. The general flow of fuzzy entropy thresholding based on the CEFO algorithm is presented in Figure 5. 

## 5. Experimental Environment

In order to verify the superiority of the CEFO algorithm in dealing with the multilevel color image segmentation problem, this section will introduce the description of our benchmark images and then select several other algorithms for comparison. The parameters of each algorithm will be described firstly and a series of quality metrics used to evaluate the quality of segmented images will be calculated at the end.

### 5.1. Benchmark Images

**Figure 6:**
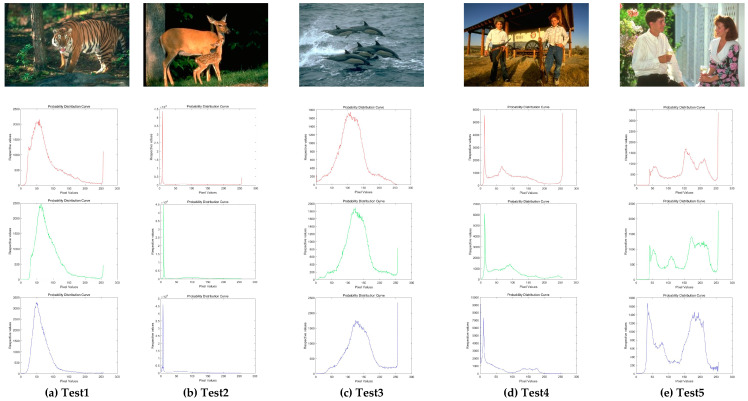

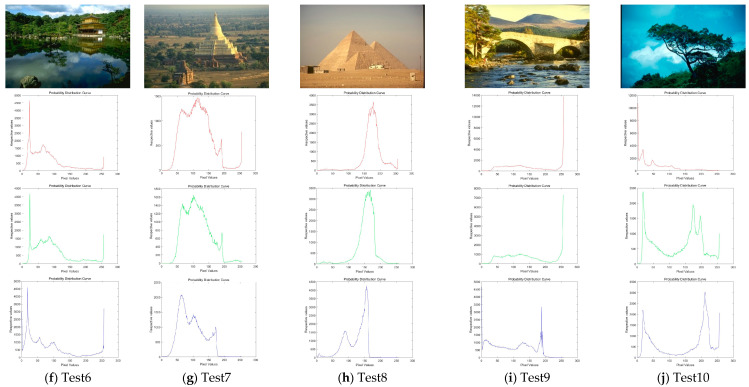
Experimental images of Berkeley. Ten classical images and their histograms of three component (red, green, and blue) are exhibited.

In this experiment, ten images are chosen from the Berkeley segmentation data set, which is shown in Figure 6. It has presented the histogram of three components about every color image. Among these images, Test 1–3 are animal images; Test 4 and 5 are about human; Test 7 and 8 are landmark buildings; Test 6 and 9 are images related to landscape architecture; Test 10 is the normal scenery image.

### 5.2. Experimental Settings

When applied to solve the problem of multilevel color image segmentation, different meta-heuristic algorithms have different optimization performances due to their strategies and mathematical formulations [43]. Therefore, it is essential to compare the CEFO algorithm with other different algorithms such as EFO, ABC [44], BA [10], BSA [19], WDO [23]. Among these algorithms, ABC, BA, and BSA are proposed from biology; EFO and WDO are inspired from physics. The number of maximum iterations of each algorithm is set to 500, and the initial population is set to 15, with a total of 30 runs per algorithm, other specific parameters are presented in Table 2.

All the algorithms are programmed in Matlab R2016a (The Mathworks Inc., Natick, MA, USA) and implemented on a Windows 7 – 64 bit with 8 GB RAM environment. 

### 5.3. Segmented Image Quality Metrics

To evaluate the quality of segmented images under different algorithms at selected threshold levels, four metrics are selected as follows [45,46]:

- Peak Signal to Noise Ratio (PSNR)

The index is used to measure the difference between the original image and the segmented image, and a higher value is gained when the segmented image has a better effect. It can be defined as:

(24) $$\begin{array}{l} PSNR(x,y)=20\cdot \log_{10}(\frac{255}{\sqrt{MSE}})\\ MSE=\frac{1}{MN}\sum_{i=0}^{M-1}\sum_{j=0}^{N-1}{\left\|x(i,j)-y(i,j)\right\|}^{2} \end{array}$$

where M and N represent the size of the image; x is the original image; y is the segmented image.

- Mean Structural Similarity (MSSIM)

The index evaluates the overall image quality, which is in the range of [−1,1]. The higher value of MSSIM is obtained when it represents the segmented image is more similar to the original image. The MSSIM is the average of every component and SSIM can be calculated as:

(25) $$SSIM(x,y)=\frac{\left(2\mu_{x}\mu_{y}+c_{1})\;(2\sigma_{xy}+c_{2}\right)}{\left(\mu_{x}^{2}+\mu_{y}^{2}+c_{1})\;(\sigma_{x}^{2}+\sigma_{y}^{2}+c_{2}\right)}$$

- Feature similarity (FSIM)

The index is in the range of [0,1], and the segmented image is better when the value is closer to 1. The FSIM can be expressed as:

(26) $$FSIM(x,y)=\frac{\sum_{x\in \Omega }S_{L}(X)PC_{m}(x)}{\sum_{x\in \Omega }PC_{m}(x)}$$

- Computation Time (CPU Time)

The index measures the convergence rate of each algorithm. The algorithm is more efficient when the time is shorter.

## 6. Results and Discussions

### 6.1. Comparison of Other Meta-Heuristic Algorithms

**Figure 7:**
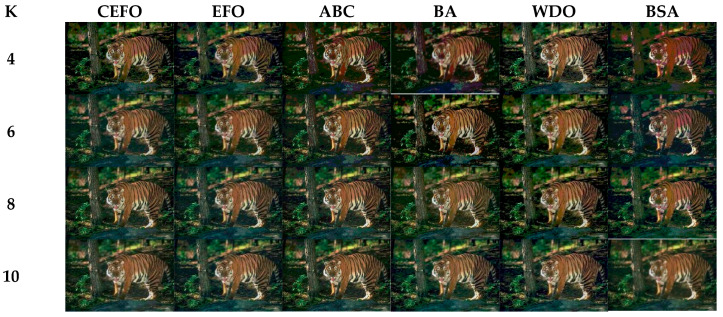
Segmented images of Test 1 at *K* = 4, 6, 8, 10 using selected algorithms based on fuzzy entropy.

**Figure 8:**
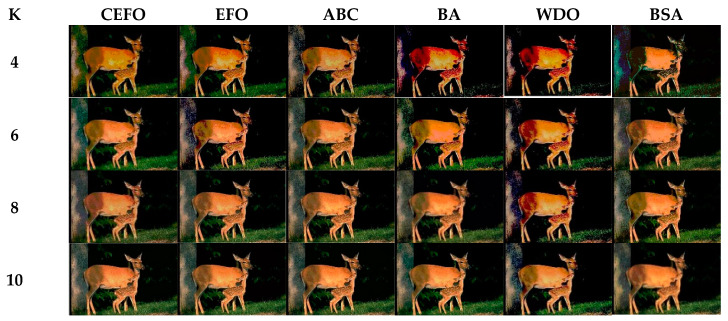
Segmented images of Test 2 at *K* = 4, 6, 8, 10 using selected algorithms based on fuzzy entropy.

**Figure 9:**
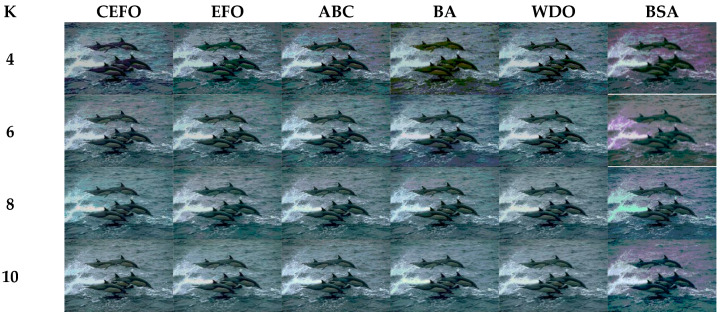
Segmented images of Test 3 at *K* = 4, 6, 8, 10 using selected algorithms based on fuzzy entropy.

**Figure 10:**
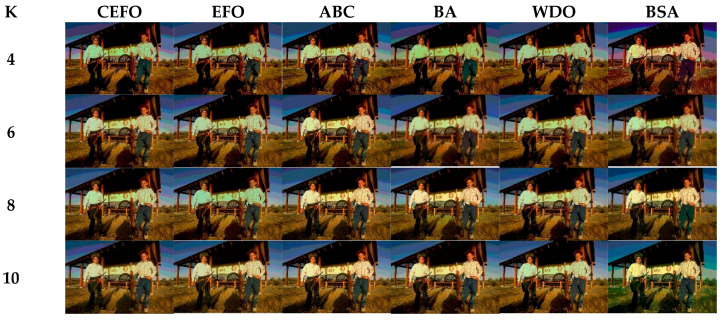
Segmented images of Test 4 at *K* = 4, 6, 8, 10 using selected algorithms based on fuzzy entropy.

**Figure 11:**
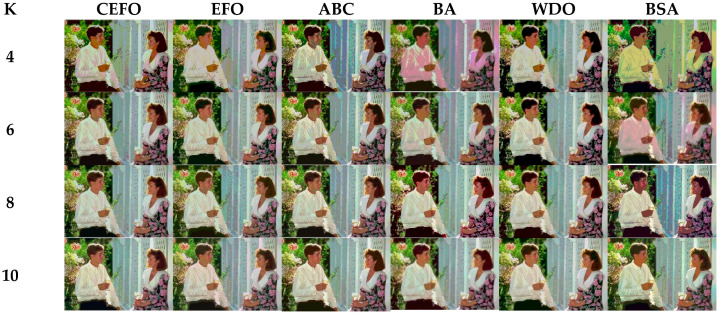
Segmented images of Test 5 at *K* = 4, 6, 8, 10 using selected algorithms based on fuzzy entropy.

**Figure 12:**
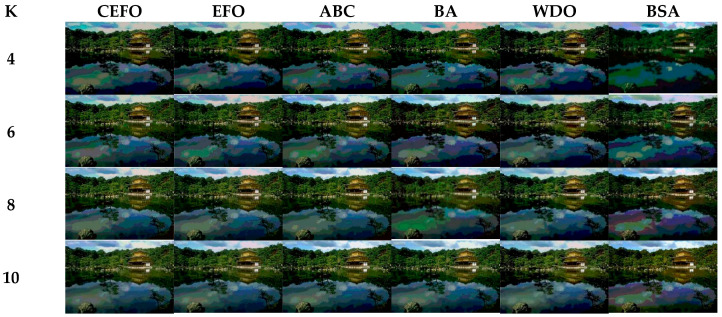
Segmented images of Test 6 at *K* = 4, 6, 8, 10 using selected algorithms based on fuzzy entropy.

**Figure 13:**
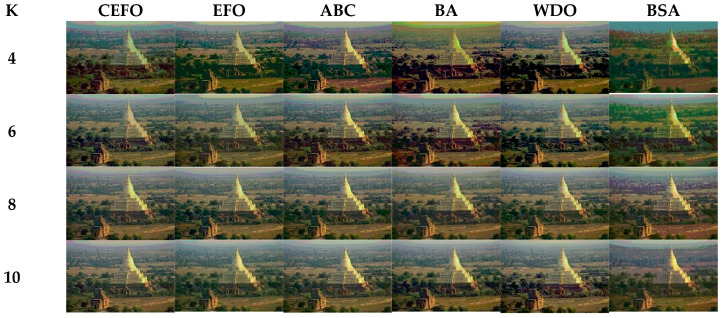
Segmented images of Test 7 at *K* = 4, 6, 8, 10 using selected algorithms based on fuzzy entropy.

**Figure 14:**
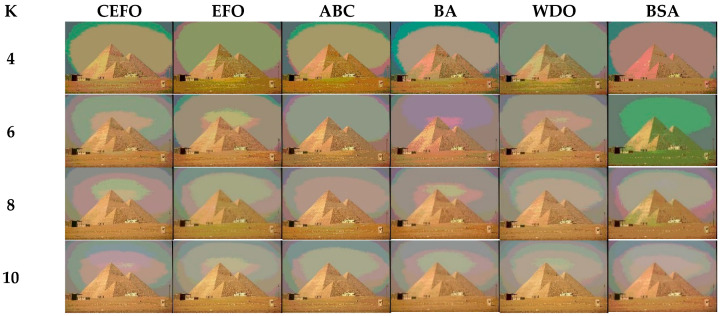
Segmented images of Test 8 at *K* = 4, 6, 8, 10 using selected algorithms based on fuzzy entropy.

**Figure 15:**
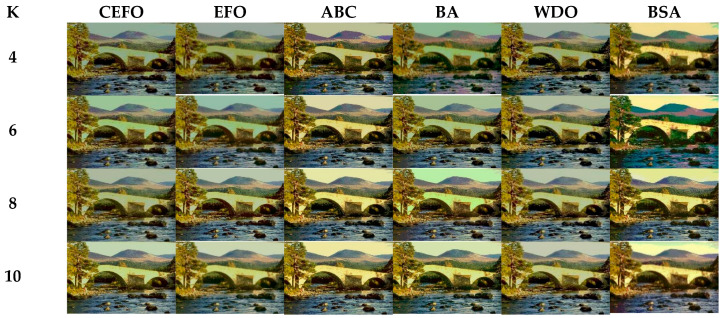
Segmented images of Test 9 at K = 4, 6, 8, 10 using selected algorithms based on fuzzy entropy.

**Figure 16:**
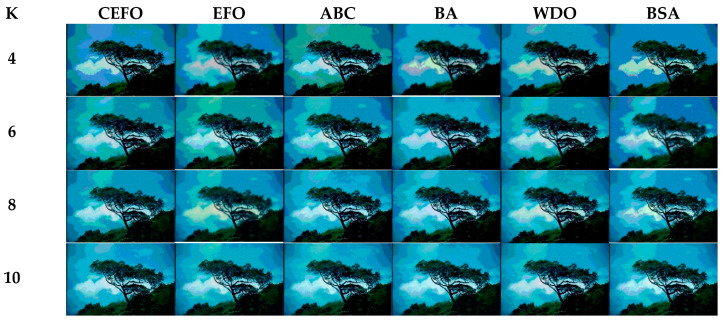
Segmented images of Test 10 at *K* = 4, 6, 8, 10 using selected algorithms based on fuzzy entropy.

Utilizing 6 algorithms based on fuzzy entropy criterion to conduct the experiment on 10 images at the threshold level of 4, 6, 8, and 10 (K = 4, 6, 8, 10). The results of the optimal threshold vector are presented in Table 3, Table 4 and Table 5 exhibiting each threshold level of three component about every image. And the results of segmented images are presented in Figure 7, Figure 8, Figure 9, Figure 10, Figure 11, Figure 12, Figure 13, Figure 14, Figure 15 and Figure 16, which take each Test image as a group. Furthermore, the results of the four metrics are shown in Table 6 and Table 7.

**Figure 17:**
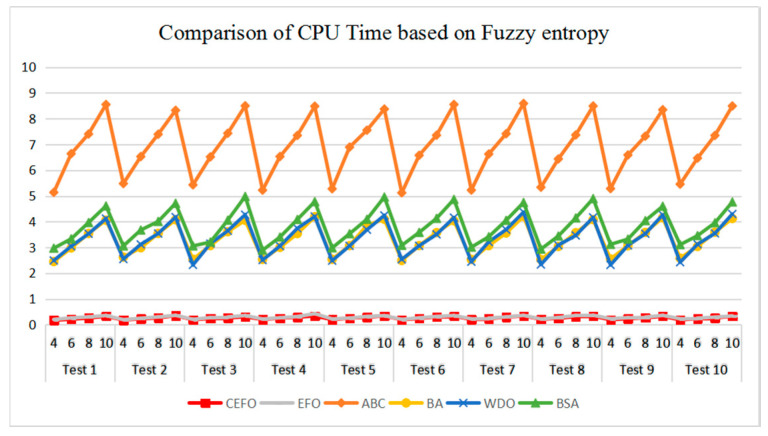
Comparison of Computational Time (CPU Time) based on fuzzy entropy.

**Figure 18:**
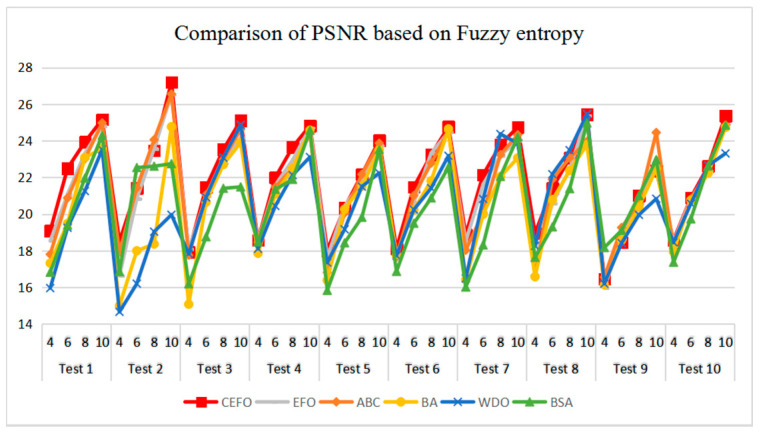
Comparison of Peak Signal to Noise Ratio (PSNR) based on fuzzy entropy.

**Figure 19:**
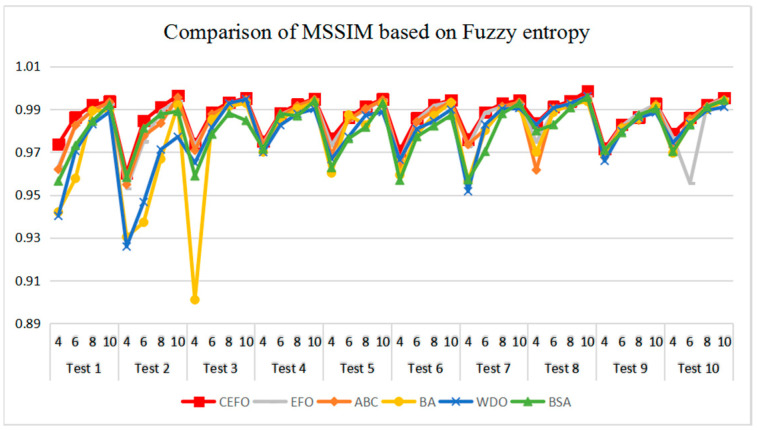
Comparison of Mean Structural Similarity (MSSIM) based on fuzzy entropy.

**Figure 20:**
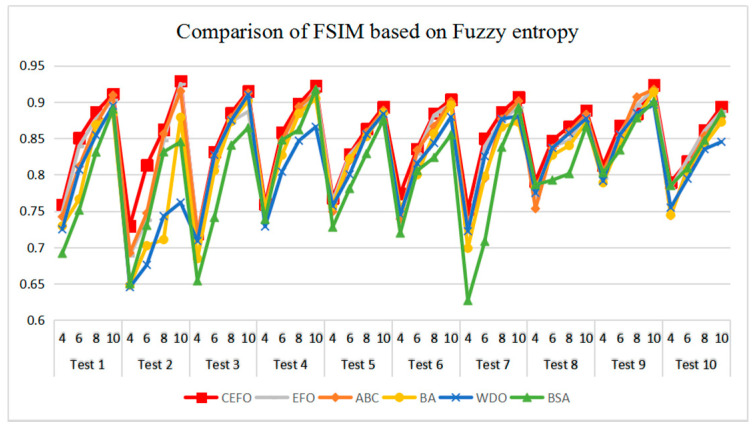
Comparison of Feature Similarity (FSIM) based on fuzzy entropy.

Table 6 and Figure 17 and Figure 18 compare the CPU Time and PSNR values while Table 7 and Figure 19 and Figure 20 compare the MSSIM and FSIM values of the segmented images. As can be seen from these tabulated values, all algorithms have lower values of PSNR, MSSIM, and FSIM at lower threshold levels. With the improvement of the threshold level, the values of PSNR, MSSIM, and FSIM increase gradually. Consequently, it can be clearly known that segmentation performance will be improved as the threshold level increases. However, the time of each algorithm will rise equally on the increasing threshold levels indicating the computation of algorithms is more complex on the higher threshold levels. PSNR, MSSIM, and FSIM are used to measure the similarity and qualify among the segmented images. Higher PSNR, MSSIM, and FSIM demonstrate that segmented images have more excellent segmentation performances. 

**Figure 21:**
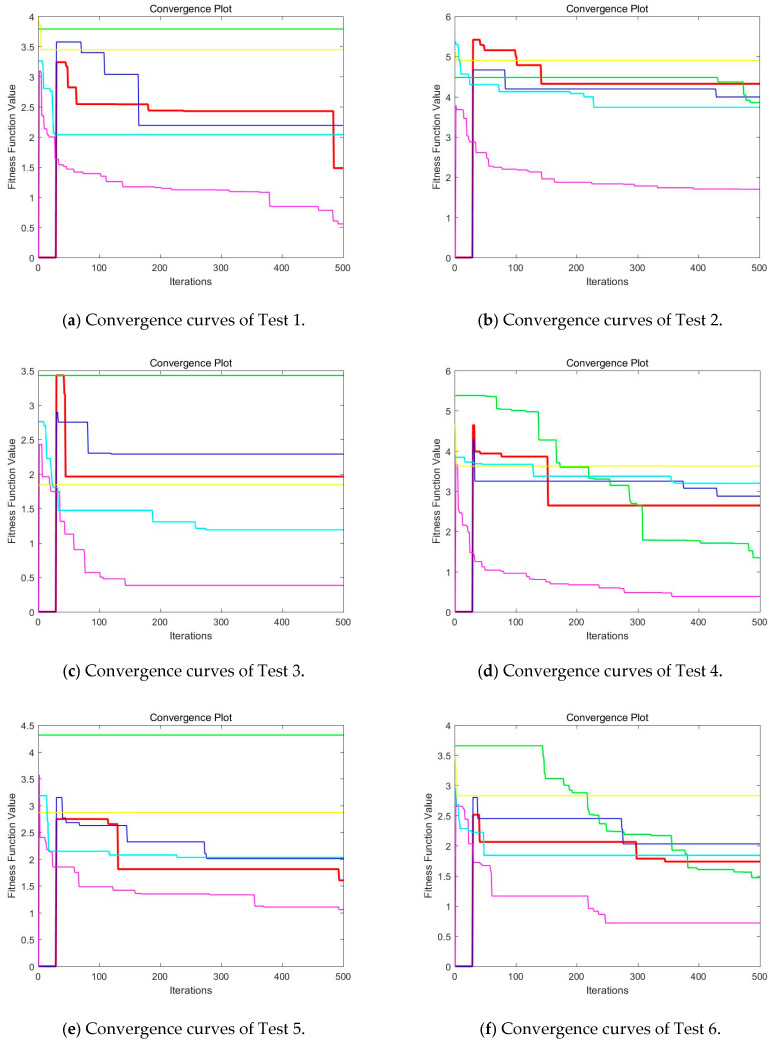

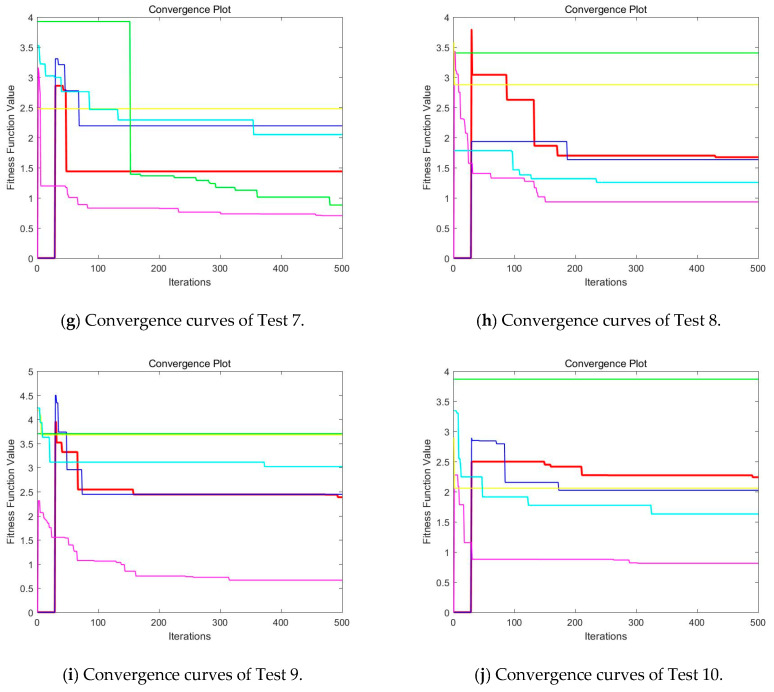
Convergence curves of CEFO, EFO, ABC, BA, WDO, and BSA based on fuzzy entropy at *K* = 10. (Red line represents CEFO; Blue line represents EFO; Cyan line represents ABC; Yellow line represents BA; Magenta line represents WDO; Green ling represents BSA.)

Then, when comparing the differences in CPU time between various algorithms, it can be found that CEFO and EFO are significantly faster than ABC, BA, WDO, and BSA. Moreover, the running time of CEFO has decreased about 12.26% when comparing with EFO, which indicates the modified electromagnetic field optimization algorithm has a faster convergence rate. As for other algorithms, ABC has the longest time of computation due to its slow convergence rate, it needs nearly 30 times as much as CEFO. Afterward, BA, WDO, and BSA have an approximate running time, they are about 15 times longer than CEFO. With the increase of execution time, the practicability of the algorithm will be reduced. For a clearer presentation of convergence speed about these algorithms, the convergence curves are shown in Figure 21.

In terms of PSNR, the chart of all algorithms is shown in Figure 18. It can be seen that CEFO has higher values among these algorithms; ABC has similar values to EFO in some images at a high threshold level. For instance, in Test 1 of K=10, PSNR value of EFO is 24.8085 while ABC is 24.9762, but CEFO is 25.1603. WDO has much lower values in smaller threshold level and BSA has good values in higher threshold level, all in all, PSNR values of BA, WDO, and BSA have different diversification, but they are mediocre on the whole. 

Comparing the results of MSSIM and FSIM in Table 7, FSIM is considered to be more authoritative and application and CEFO also performs better than other algorithms in this index. Although EFO can have a banner performance at *K* = 4, ABC will usually be close to EFO at high levels. BA has better values at some images such as in Test 2, 4, etc. WDO and BSA have higher values than BA at *K* = 6 in some images but they are all lower than CEFO on the whole.

From what has been mentioned above, the CEFO algorithm has superior performance when searching for the optimal threshold vector in multilevel thresholding color image segmentation.

### 6.2. Comparison of Other Segmentation Methods

**Figure 22:**
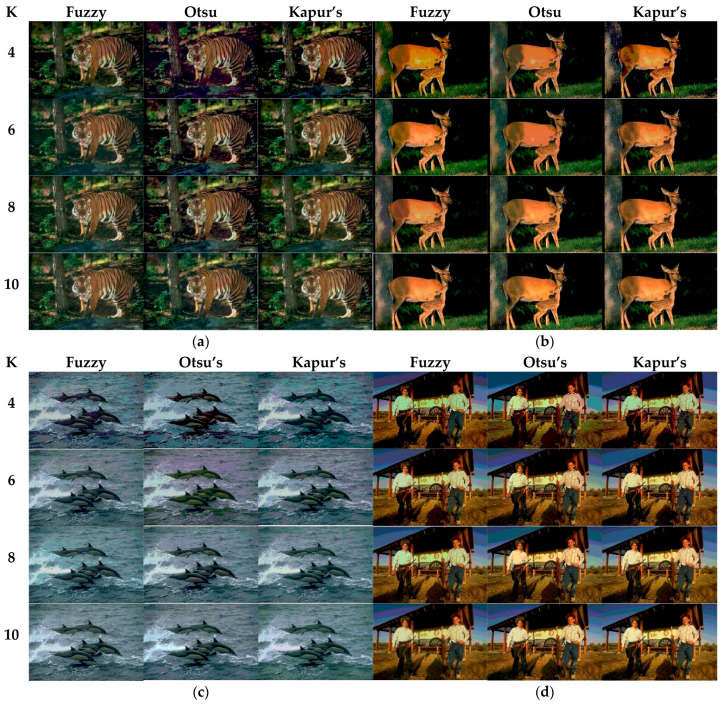

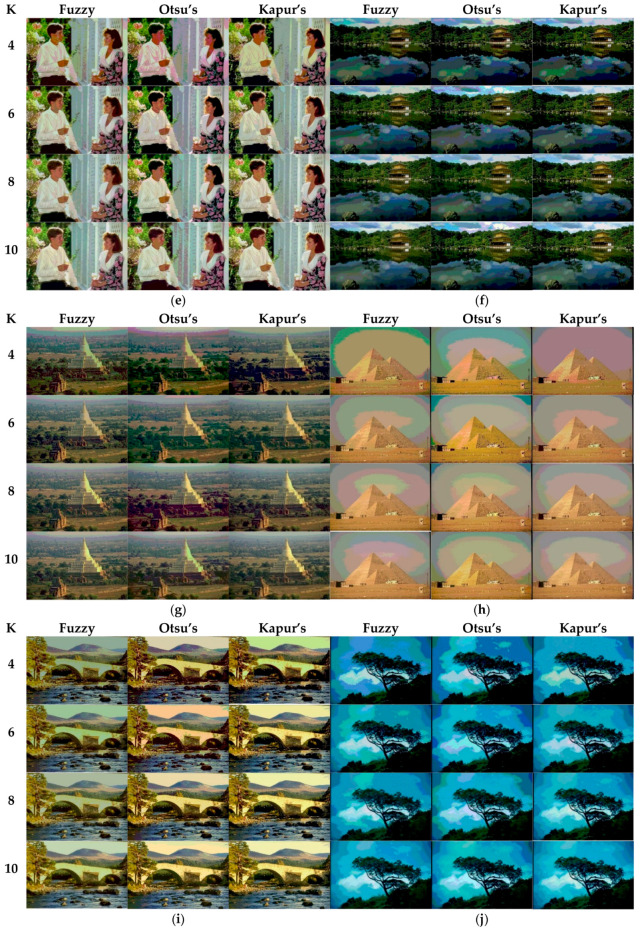
Segmented images at *K* = 4, 6, 8, 10 using CEFO algorithm based on fuzzy entropy, Otsu’s and Kapur’s entropy.

In the last experiment, the superiority of CEFO has been verified. And in this experiment, fuzzy entropy has been regarded as the research objective. To show the performance of fuzzy entropy thresholding in multilevel color image segmentation, Otsu’s and Kapur’s entropy based on color image segmentation are used to be a comparison. Applying the CEFO algorithm to Fuzzy entropy, Otsu’s, and Kapur’s entropy respectively to segment selected 10 Berkeley images in Figure 6. The threshold level is chosen as = 4, 6, 8, and 10, which is used to obtain the corresponding threshold points for each component of the color image. The results of segmented images are in Figure 22, and the corresponding optimal threshold values are in Table 8. Table 9 compares the performance of different thresholding approaches based on parameters of CPU Time, PSNR, MSSIM, and FSIM.

**Figure 23:**
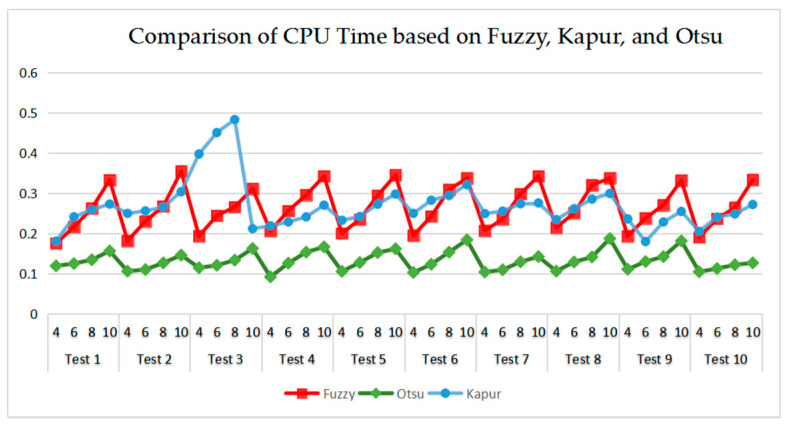
Comparison of CPU Time based on fuzzy entropy.

**Figure 24:**
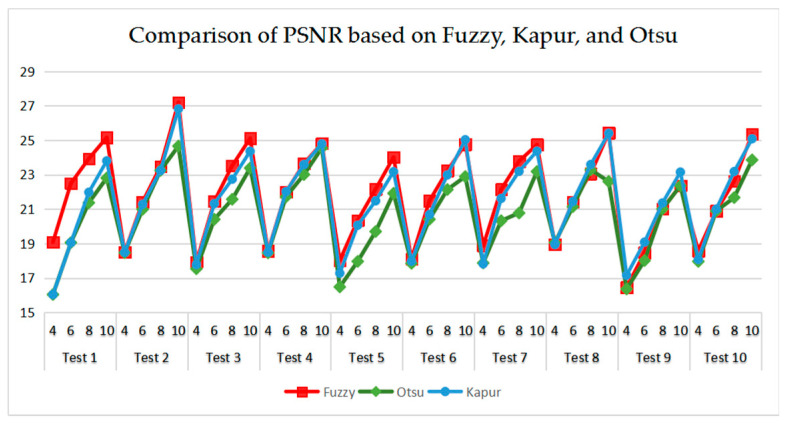
Comparison of PSNR based on fuzzy entropy.

**Figure 25:**
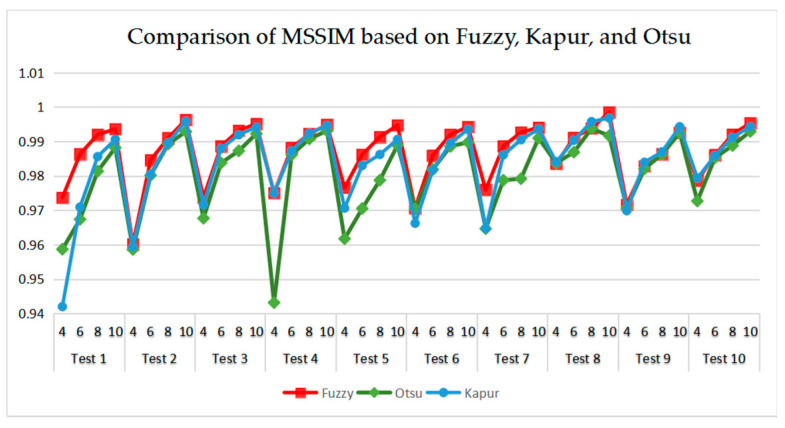
Comparison of MSSIM based on fuzzy entropy.

**Figure 26:**
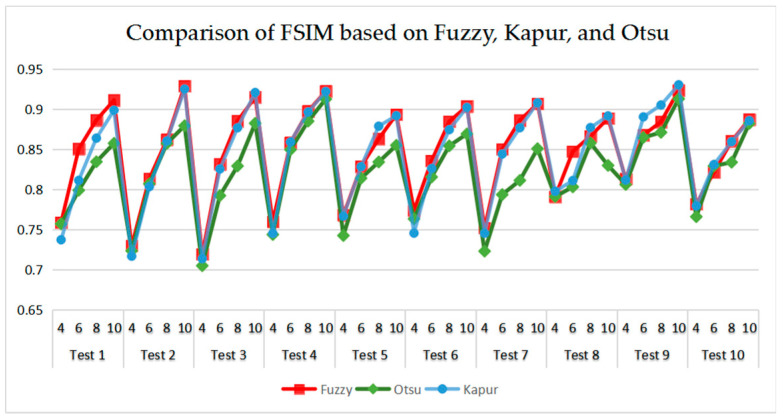
Comparison of FSIM based on fuzzy entropy.

As can be seen in Table 9 and Figure 23, Figure 24, Figure 25 and Figure 26, Otsu’s thresholding has the fastest speed of execution time, fuzzy entropy and Kapur’s entropy are a bit slower than Otsu’s. However, three thresholding segmentation methods are all in 0.5 (seconds) at different threshold levels, which can also indicate CEFO algorithm has a fast convergence rate. In terms of PSNR, it is clear that fuzzy entropy thresholding has higher values in general, the ranking of PSNR among these segmentation methods is Fuzzy > Kapur’s > Otsu’s. As for MSSIM and FSIM, Fuzzy entropy also performs well, which is in advance of two other methods overall. And the ranking of MSSIM and FSIM among three methods is Fuzzy > Kapur’s > Otsu’s. Therefore, fuzzy entropy is better as compared to others showing CEFO based on the fuzzy entropy technique can be applied in the color image segmentation field excellently.

### 6.3. ANOVA Test

A statistical test known as “the analysis of variance” (ANOVA) has been performed at 5% significance level to evaluate the significant difference between algorithms. In the experiment, CEFO algorithm is regarded as the control group and is compared with EFO, ABC, BA, WDO and BSA algorithms in terms of four measure metrics. The null hypothesis assumes that there is no significant difference between the mean values of 5 selected algorithms, whereas, the alternative hypothesis can be considered as a significant difference between them. Table 10 exhibits the –value of CPU Time, PSNR, MSSIM, and FSIM by the ANOVA test. As can be seen, the -value for CPU Time is less than 0.05, which implies a significant difference between the proposed algorithm and other algorithms and CEFO has a much fast convergence rate. With respect to another three measures, CEFO also has significant difference about BA, WDO and BSA. It can be observed that CEFO algorithm has a better performance. 

## 7. Conclusions and Future Work

In this paper, multilevel thresholding color image segmentation has been considered as an optimization problem in which the fuzzy entropy technique has been presented as the objective function. To achieve efficient segmentation, it is essential for algorithms to search the optimal fuzzy parameters and threshold values. Electromagnetic Field Optimization is a novel meta-heuristic algorithm which use is attempt herein for the first time in this field. Additionally, a new chaotic strategy is proposed and embedded into the EFO algorithm to accelerate the convergence rate and enhance segmentation accuracy. In order to demonstrate the superior performance of the CEFO-based fuzzy entropy technique, a series of experiments have been conducted and results are evaluated in terms of CPU Time, PSNR, MSSIM, and FSIM. On the one hand, the CEFO algorithm is compared with EFO, ABC, BA, WDO, and BSA based on fuzzy entropy for segmenting ten Berkeley benchmark images at different threshold levels (*K* = 4, 6, 8, and 10). The obtained results illustrate the obvious effect of proposed chaotic strategy and CEFO needs less than 0.35 seconds to find the optimal threshold vector which makes it an effective algorithm to handle the above problem. On the other hand, the fuzzy entropy method is compared with Otsu’s variance and Kapur’s entropy method based on CEFO on the basis of the same experimental environment. The high precision of fuzzy entropy has been validated with four metrics. Although CEFO-fuzzy is not the fastest among the three techniques, its execution time is suitable for practical applications within 0.5 seconds. To sum up, CEFO-based fuzzy entropy is a robust technique in multi-threshold color image segmentation. 

In the future, the proposed technique can be applied to solve practical problems such as medical images, satellite images, etc. It is also interesting to modify EFO algorithm in other aspects to improve its performance for higher threshold levels (e.g., *K* = 15 and 20). Furthermore, the merits of CEFO can be investigated using Tsallis entropy, Renyi’s entropy, and cross entropy for multilevel thresholding.

## Figures and Tables

**Table 1:** Chaotic maps.

Name	Chaotic Map
Logistic	$x_{i+1}=ax_{i}(1-x_{i})$
Sine	$x_{i+1}=\frac{a}{4}\sin(\pi x_{i})$
Cubic	$x_{i+1}=ax_{i}(1-x_{i}^{2})$
Circle	$x_{i+1}=mod(x_{i}+b-(\frac{a}{2\pi })\sin(2\pi x_{i}^{}),1)$
Iterative	$x_{i+1}=\sin(\frac{a\pi }{x_{i}})$
Tent	$x_{i+1}=a-(1-a)\left|x_{i}\right|$

**Table 2:** Specific values of parameters used in selected algorithms.

Algorithm	Parameter	Explanation	Value
EFO	*P_field_*	The portion of particles belonging to the positive field.	0.1
	*N_field_*	The portion of particles belonging to the negative field.	0.45
	*Ps_field_*	The probability of selecting electromagnets directly from the positive field.	0.3
	*R_rate_*	The probability of changing one electromagnet directly from the positive field.	0.2
ABC	*limit*	The value of the max trial limit.	10
BA	*r_i_*	The rate of pulse emission.	[0, 1]
	*A_i_*	The value of loudness.	[1, 2]
WDO	a	A constant.	0.4
	*g*	The constant of gravitation.	0.2
	*RT*	A coefficient.	3
	c	Coriolis coefficient.	0.4
BSA	*a*_1_, *a*_2_	The values of indirect and direct effects on the birds’ vigilance behaviors.	2
	*c*_1_, *c*_2_	The values of the cognitive coefficient and social coefficient.	2
	*FL*	The frequency of birds’ flight behaviors.	0.6

**Table 3:** Comparison of optimal threshold values between Chaotic Electromagnetic Field Optimization (CEFO) and Electromagnetic Field Optimization (EFO) at *K* = 4, 6, 8, 10 based on fuzzy entropy.

Image	K	CEFO			EFO		
		R	G	B	R	G	B
**Test 1**	**4**	56 93 153 187	57 88 132 191	59 10 157 197	61 90 144 188	18 74 115 170	58 87 148 202
	**6**	20 59 92 128 167 199	26 47 81 114 161 203	25 48 76 111 138 195	26 60 95 128 162 198	19 47 80 119 171 210	18 57 85 122 154 212
	**8**	11 31 58 82 107 132 168 211	12 42 64 92 114 149 181 210	18 50 73 97 143 168 196 223	17 47 71 90 115 138 171 209	12 39 67 93 130 170 205 223	26 56 78 100 113 162 190 216
	**10**	14 37 55 75 90 111 130 159 182 210	11 41 61 95 122 146 168 185 208 237	14 40 64 80 105 136 161 189 212 231	10 26 56 82 102 133 148 167 192 215	11 32 59 76 90 107 134 166 198 222	24 41 56 72 93 126 156 181 203 229
**Test 2**	**4**	10 93 114 171	86 143 194 226	91 110 137 189	80 116 149 183	93 113 146 216	104 124 170 206
	**6**	18 41 75 107 147 177	87 101 133 153 192 234	54 72 97 121 156 192	73 83 108 139 176 219	94 114 135 162 197 231	16 50 85 129 156 199
	**8**	15 27 44 59 96 127 171 204	14 22 43 65 117 144 190 222	39 61 82 117 148 176 195 223	7 34 55 78 107 135 166 199	49 65 76 139 168 193 211 232	45 61 82 101 129 154 182 213
	**10**	32 55 75 108 144 171 195 215 226 239	5 50 65 85 102 121 139 167 197 228	31 47 64 87 108 125 158 179 209 239	4 24 47 67 102 122 147 180 204 223	19 33 53 71 99 119 144 170 195 224	19 36 57 80 108 141 154 177 201 230
**Test 3**	**4**	44 92 139 188	31 92 139 188	27 97 138 185	33 80 126 181	37 87 142 206	29 106 154 188
	**6**	32 62 93 128 173 199	19 53 90 119 162 206	36 71 105 136 183 208	32 76 116 150 177 207	50 69 101 143 190 216	15 46 70 113 153 197
	**8**	22 43 68 91 112 138 172 208	17 51 73 98 123 148 181 207	11 36 58 88 106 141 170 213	19 54 94 125 141 164 190 214	25 49 72 96 126 156 192 223	15 49 71 95 128 159 187 214
	**10**	26 39 55 69 86 110 131 154 175 207	42 29 56 79 104 121 141 163 197 219	9 25 49 82 109 132 154 176 199 222	17 31 45 62 93 124 168 194 212 229	14 39 56 76 95 114 148 176 196 224	16 13 34 58 82 115 150 180 199 223
**Test 4**	**4**	52 84 127 164	67 102 141 204	49 74 111 155	59 88 129 164	64 105 153 199	61 101 145 203
	**6**	38 62 97 137 172 195	48 71 111 144 175 213	43 77 109 147 178 235	40 65 89 112 147 184	44 67 105 126 167 211	39 63 98 134 160 190
	**8**	13 31 54 92 110 142 175 198	35 60 86 114 137 169 200 226	24 41 64 94 129 156 193 224	15 26 46 68 97 134 160 189	18 43 73 100 128 155 190 225	23 57 105 137 169 195 221 237
	**10**	6 25 43 64 84 106 132 152 172 189	7 27 39 52 75 99 130 157 190 221	15 25 48 78 103 130 157 178 219 240	8 18 31 48 74 100 124 153 181 211	8 23 39 57 80 104 127 157 188 215	10 27 50 87 117 146 171 194 218 234
**Test 5**	**4**	52 98 149 198	55 89 164 215	77 125 162 209	87 122 152 190	38 58 106 162	80 120 160 194
	**6**	22 63 93 131 179 225	16 69 97 125 158 204	15 61 97 131 166 207	23 64 90 140 183 213	26 75 114 134 169 211	20 59 84 132 169 205
	**8**	16 59 77 94 116 134 162 199	20 58 75 98 129 155 185 214	19 56 77 115 145 173 196 227	14 56 75 104 127 162 191 225	37 37 60 76 111 135 175 214	17 39 66 97 122 171 205 234
	**10**	13 29 57 77 93 107 130 165 188 214	14 30 62 78 94 126 152 179 205 231	19 44 56 72 89 109 129 159 176 205	11 26 50 72 92 115 149 180 201 224	16 25 48 64 85 106 127 142 175 206	24 41 56 70 96 123 150 177 202 229
**Test 6**	**4**	55 97 158 195	64 99 153 190	46 75 131 179	52 100 144 192	56 92 123 178	52 88 149 176
	**6**	43 60 91 118 165 202	25 38 76 119 165 205	47 69 97 133 155 190	44 75 112 150 176 211	60 87 109 135 167 196	32 51 85 125 172 207
	**8**	16 42 73 91 129 157 190 221	10 34 52 79 108 136 168 207	23 39 64 86 120 137 172 202	28 45 74 91 120 150 191 225	15 26 55 85 111 141 176 205	23 38 57 87 119 159 183 214
	**10**	10 32 50 77 99 134 162 192 209 228	13 36 47 69 107 140 164 185 200 223	14 33 60 82 101 130 156 183 208 228	8 30 46 72 99 125 145 166 189 221	10 28 53 72 100 125 146 172 187 206	14 31 51 68 86 115 134 167 190 221
**Test 7**	**4**	66 95 139 176	25 66 108 157	23 88 134 220	26 89 132 179	43 89 133 178	30 94 137 208
	**6**	17 33 80 120 168 205	20 80 108 133 164 189	13 59 84 102 128 151	20 41 60 92 131 180	21 54 86 111 144 183	31 67 101 138 196 223
	**8**	20 54 79 106 126 146 169 209	13 50 80 102 132 166 197 223	19 40 63 88 119 148 200 228	14 58 80 98 122 146 167 207	18 55 82 105 128 161 193 228	18 36 67 91 128 149 200 220
	**10**	10 30 41 63 89 110 129 154 178 216	14 54 68 82 106 132 169 197 220 243	15 41 66 85 105 133 151 190 214 233	12 28 59 82 115 146 179 199 215 227	12 31 57 76 92 117 141 168 200 223	10 34 63 95 120 140 157 185 212 232
**Test 8**	**4**	48 73 173 212	62 107 141 207	43 79 124 215	22 83 139 199	55 105 149 201	37 102 183 234
	**6**	38 64 101 141 181 224	32 61 85 126 155 196	38 61 91 126 197 218	40 66 96 151 182 212	40 66 97 140 175 209	34 54 88 117 182 230
	**8**	15 41 64 85 110 133 175 213	44 34 74 110 135 158 179 203	25 40 69 104 136 182 207 231	31 55 77 93 108 135 178 217	25 51 80 117 155 179 206 227	30 32 60 103 117 141 179 215
	**10**	18 39 58 87 115 137 172 186 210 230	21 36 63 79 111 129 150 180 208 227	28 47 74 90 116 131 153 178 208 226	22 42 69 84 109 133 158 182 208 227	39 91 103 127 146 159 176 193 208 225	21 35 65 98 119 140 165 184 211 231
**Test 9**	**4**	47 74 107 149	55 90 132 167	42 66 117 149	33 67 109 155	53 87 123 165	45 89 155 211
	**6**	13 43 62 92 126 165	15 50 89 125 160 195	19 45 81 115 151 230	12 42 59 96 127 160	24 33 76 115 143 190	28 48 80 116 149 167
	**8**	14 36 53 75 92 123 152 173	11 40 55 77 101 126 151 183	26 41 66 91 128 155 200 233	11 38 54 81 115 139 166 187	17 66 86 105 125 145 172 197	28 51 76 104 132 154 172 207
	**10**	13 23 41 59 82 106 126 158 181 198	11 43 54 72 89 105 134 163 188 213	24 47 71 94 118 140 160 177 208 237	9 41 60 92 116 131 150 167 185 203	13 30 47 67 86 108 131 150 183 212	20 43 71 92 115 155 175 190 226 240
**Test 10**	**4**	48 76 132 175	31 69 117 169	53 86 116 200	62 101 132 190	45 70 123 186	52 83 139 193
	**6**	34 57 90 127 166 196	53 90 116 142 180 214	42 56 104 136 169 220	33 56 87 127 162 198	43 76 118 155 190 225	42 70 100 133 164 226
	**8**	19 33 62 91 133 165 195 214	20 26 57 90 112 150 195 220	12 38 55 88 113 150 186 229	34 60 98 128 148 170 194 221	17 38 58 87 119 147 187 216	10 23 49 79 121 149 170 189
	**10**	16 35 54 71 93 119 141 169 196 231	9 26 36 51 72 106 132 166 195 224	10 26 41 61 77 108 134 160 185 213	14 33 56 86 106 129 159 189 212 235	8 29 43 75 99 118 141 168 196 226	10 33 48 69 90 113 144 169 194 230

**Table 4:** Comparison of optimal threshold values between Artificial Bee Colony (ABC) and Bat Algorithm (BA) at *K* = 4, 6, 8, 10 based on fuzzy entropy.

Image	K	ABC			BA		
		R	G	B	R	G	B
**Test 1**	**4**	55 103 154 201	69 104 156 207	40 96 165 219	49 78 150 214	66 92 128 195	78 116 176 213
	**6**	24 63 109 149 184 220	47 74 111 152 183 218	39 84 126 154 188 220	31 58 81 105 150 195	20 45 85 115 165 212	29 71 118 154 190 230
	**8**	24 51 81 113 142 177 211 226	20 54 77 108 150 174 200 223	15 47 75 107 143 171 207 237	12 51 80 98 117 155 193 225	13 48 77 109 159 181 205 225	33 63 94 110 143 168 203 242
	**10**	16 41 59 85 113 142 167 187 211 236	15 43 62 88 112 139 167 189 209 232	13 39 62 83 113 139 165 192 221 236	20 50 82 99 125 149 168 190 211 231	15 41 88 107 129 146 158 177 206 232	31 51 72 84 99 129 162 191 227 242
**Test 2**	**4**	63 108 164 216	78 119 159 231	72 98 171 224	117 150 188 275	129 152 182 231	88 113 140 189
	**6**	49 76 112 149 196 236	52 79 125 159 189 223	56 78 124 148 182 219	81 107 143 173 205 232	83 97 112 130 212 233	83 114 153 178 206 238
	**8**	38 51 73 110 143 182 221 240	37 56 90 122 143 170 195 222	31 51 79 109 149 175 207 246	35 48 68 89 140 181 210 236	51 85 120 156 182 214 242 250	5 20 45 82 113 149 177 217
	**10**	22 37 57 85 112 142 167 192 223 237	6 45 60 80 109 133 160 188 218 244	23 40 65 88 115 144 163 188 218 239	64 69 75 81 98 120 155 187 210 230	30 50 70 92 120 147 171 193 214 238	28 58 79 98 135 162 186 206 234 248
**Test 3**	**4**	31 103 162 206	60 102 151 209	44 103 157 206	53 99 141 197	53 121 164 203	23 100 143 191
	**6**	34 68 103 140 190 226	29 66 101 140 186 217	31 67 98 138 186 217	29 66 116 154 185 215	39 72 122 154 181 210	45 86 116 143 173 219
	**8**	18 51 79 111 138 173 205 232	28 56 84 108 140 168 194 231	20 50 77 103 141 168 199 222	20 43 75 97 136 181 205 236	25 51 80 108 136 177 203 231	12 56 86 110 138 175 202 227
	**10**	19 37 63 87 112 137 170 195 222 244	22 46 65 92 118 141 165 194 216 237	21 52 71 96 110 136 157 196 214 230	19 40 64 90 117 147 174 206 227 244	36 62 88 105 116 135 162 196 216 239	30 51 74 100 113 135 146 160 184 214
**Test 4**	**4**	40 90 136 172	63 118 170 210	63 96 146 208	68 110 141 168	60 112 161 208	49 109 189 234
	**6**	32 69 114 155 190 219	42 66 111 161 201 237	35 65 104 146 186 235	38 59 116 147 173 197	37 78 135 168 193 223	40 74 108 137 155 226
	**8**	21 45 78 107 137 169 206 227	32 56 83 120 142 177 212 241	26 49 80 116 156 182 213 233	8 36 61 92 125 197 222 241	26 52 84 112 135 160 198 228	26 58 91 121 146 170 198 228
	**10**	25 45 60 84 114 140 168 191 217 232	26 43 62 86 108 136 161 191 216 245	22 38 63 85 113 137 159 193 223 241	8 33 64 96 128 156 174 195 217 234	38 63 84 105 129 145 168 192 206 231	25 41 54 67 96 130 152 186 209 233
**Test 5**	**4**	34 94 167 222	29 93 159 220	75 118 169 231	33 107 160 196	29 75 134 225	72 106 149 208
	**6**	26 72 97 138 187 223	21 73 108 140 188 226	20 71 118 153 191 231	59 86 122 163 202 228	19 62 94 144 184 234	52 101 136 176 213 234
	**8**	16 52 84 117 141 175 202 229	13 62 86 117 143 178 203 228	18 56 77 103 135 172 200 234	27 61 85 117 142 171 201 238	89 124 141 157 171 183 198 225	56 78 98 126 157 167 199 234
	**10**	18 36 68 99 124 147 167 183 206 228	12 50 68 88 114 144 169 192 219 238	18 41 65 92 111 133 163 190 220 241	17 37 54 72 104 138 160 178 191 231	14 68 86 104 118 141 168 197 216 231	16 43 68 98 128 154 177 198 220 241
**Test 6**	**4**	72 116 162 197	59 92 142 177	60 91 143 196	60 121 178 210	55 91 135 174	59 94 128 166
	**6**	35 54 103 142 184 222	31 66 111 161 192 228	36 74 114 158 197 218	12 59 96 121 156 201	61 110 147 175 204 233	44 74 103 131 190 227
	**8**	16 45 79 113 151 189 210 231	13 51 85 119 156 182 212 234	21 44 84 120 148 176 206 224	13 33 48 60 119 152 190 236	41 64 98 119 158 183 203 222	11 40 62 125 165 183 213 234
	**10**	15 42 60 86 114 144 164 194 214 231	11 41 60 81 101 130 155 181 212 233	26 46 64 80 110 131 164 185 201 220	11 47 75 102 121 134 150 178 204 227	14 39 58 84 108 138 161 186 210 235	21 38 51 71 90 115 144 172 191 228
**Test 7**	**4**	55 97 147 210	45 96 148 216	41 92 139 220	67 96 125 171	84 112 163 244	24 76 173 219
	**6**	33 72 111 153 177 219	19 75 110 147 181 227	25 71 109 148 190 227	57 75 92 122 166 212	27 87 118 141 185 234	21 67 100 140 192 235
	**8**	24 59 79 102 129 157 188 221	29 65 87 113 145 166 195 231	24 56 86 113 147 189 211 234	41 72 92 116 138 154 169 212	22 45 79 104 133 162 200 226	14 45 72 111 154 188 212 244
	**10**	15 39 68 98 125 142 166 195 220 237	13 40 69 87 110 140 170 202 223 242	17 44 71 89 113 138 158 193 214 234	21 62 94 122 144 163 183 200 217 242	43 80 102 121 140 159 175 196 223 237	20 50 84 111 131 167 180 205 223 239
**Test 8**	**4**	55 86 183 220	60 104 153 225	33 85 121 226	32 78 163 226	63 106 148 222	54 97 126 192
	**6**	38 82 126 154 193 229	44 74 114 152 181 230	35 57 76 105 130 235	46 87 148 181 215 237	53 78 119 144 184 227	27 42 65 90 112 141
	**8**	14 48 81 105 142 175 212 239	24 55 90 116 146 174 208 236	29 62 80 110 133 172 208 236	28 71 98 124 149 179 204 233	24 54 81 104 142 177 211 236	9 21 42 84 113 132 192 240
	**10**	11 34 57 78 101 133 167 191 216 234	17 35 64 85 109 134 155 183 210 239	20 40 72 99 116 135 167 186 218 240	16 51 79 103 126 144 169 194 224 239	18 50 66 83 101 121 147 172 207 227	18 47 82 105 127 145 169 194 220 240
**Test 9**	**4**	64 110 138 182	43 96 149 194	52 101 145 221	68 104 122 151	35 73 109 182	38 92 144 208
	**6**	19 62 101 131 157 197	39 74 106 145 183 212	30 65 102 135 166 234	39 63 90 116 141 181	12 51 100 129 161 198	40 54 72 101 129 158
	**8**	21 56 98 134 154 173 194 214	16 43 67 99 141 175 197 216	22 50 83 115 146 168 205 239	38 58 74 104 128 145 175 198	40 60 105 141 163 187 213 236	15 29 60 96 126 167 210 239
	**10**	12 42 63 87 114 141 169 197 223 238	15 46 66 86 110 137 160 187 214 236	25 45 60 85 111 136 164 193 227 242	23 66 88 123 142 154 164 179 199 216	14 42 64 84 111 135 159 184 203 226	16 32 48 99 135 158 174 202 227 243
**Test 10**	**4**	58 104 164 221	70 110 161 213	77 122 159 215	74 134 165 199	70 109 164 238	78 104 163 193
	**6**	38 76 125 159 192 229	53 79 111 139 176 218	48 71 101 135 182 221	26 80 137 166 196 233	51 96 118 138 169 209	42 74 121 155 198 229
	**8**	31 49 76 110 142 169 202 240	32 52 86 117 148 180 206 226	29 52 83 114 146 171 195 234	32 67 94 119 147 174 199 240	47 75 90 103 117 159 187 219	35 67 108 136 162 185 215 243
	**10**	27 47 69 94 120 143 164 185 212 242	26 43 66 95 117 142 165 192 221 239	22 39 62 89 110 135 157 185 214 238	33 66 88 111 125 143 156 180 201 239	11 29 53 84 114 136 166 193 220 234	13 39 58 105 132 166 183 207 230 247

**Table 5:** Comparison of optimal threshold values between Wind Driven Optimization (WDO) and Bird Swarm Algorithm (BSA) at *K* = 4, 6, 8, 10 based on fuzzy entropy.

Image	K	WDO			BSA		
		R	G	B	R	G	B
**Test 1**	**4**	68 107 150 192	65 103 156 198	77 115 164 196	54 119 190 243	12 57 174 243	24 93 175 238
	**6**	44 74 109 134 172 205	53 84 120 149 175 205	61 95 131 154 186 218	22 90 100 165 239 255	6 30 78 158 214 239	35 58 93 153 186 227
	**8**	47 72 101 121 144 168 188 211	48 75 104 129 149 170 192 213	46 68 91 110 131 156 174 212	7 57 108 118 129 158 216 251	9 55 68 97 181 209 241 255	4 32 78 123 152 179 196 247
	**10**	45 67 93 115 131 142 161 176 194 210	51 65 82 98 114 133 152 171 195 217	25 45 65 80 95 113 139 164 194 223	12 27 46 70 102 136 153 178 198 228	17 37 49 72 95 132 155 194 213 226	12 33 63 99 132 161 189 212 228 243
**Test 2**	**4**	123 146 174 202	128 154 183 216	98 131 170 208	7 39 194 232	24 112 145 227	71 105 128 183
	**6**	100 117 138 160 185 205	109 129 153 171 195 225	87 103 128 164 181 214	17 51 79 129 191 230	3 18 55 104 145 223	26 70 90 142 217 240
	**8**	78 93 104 118 134 146 169 200	93 111 131 147 165 182 202 228	62 74 92 111 131 154 179 211	17 52 83 123 181 216 230 247	18 73 92 119 152 190 225 252	59 90 102 121 142 198 224 240
	**10**	70 82 95 107 122 134 150 168 189 209	80 90 101 112 120 130 142 157 170 190	70 83 103 118 137 147 166 182 200 218	1 20 75 79 82 87 118 142 179 211	16 38 73 114 130 163 196 248 253 255	22 73 90 123 137 148 179 205 215 239
**Test 3**	**4**	48 104 158 199	68 121 163 196	77 119 161 201	39 94 147 214	42 117 173 201	24 107 208 221
	**6**	44 80 113 148 176 212	47 77 105 141 180 214	56 83 114 136 168 211	62 96 128 176 199 223	16 42 63 76 122 219	12 34 67 126 183 212
	**8**	37 67 92 112 135 163 189 217	38 59 82 102 129 156 188 213	46 70 91 117 140 162 190 213	1 37 51 77 115 159 209 237	19 60 102 149 177 203 223 251	20 57 73 95 125 159 188 221
	**10**	36 69 100 120 142 156 172 190 204 223	30 54 73 96 119 137 158 178 198 222	24 49 67 83 104 120 143 170 192 214	8 77 109 140 164 179 202 207 231 254	1 25 82 98 109 164 178 196 230 255	17 56 80 97 119 146 172 216 229 251
**Test 4**	**4**	54 94 132 169	71 117 154 200	78 109 147 207	46 89 145 214	18 95 184 244	36 77 130 213
	**6**	49 72 101 128 156 183	58 87 109 143 181 212	51 89 115 152 194 223	35 52 85 128 167 193	41 70 93 146 203 231	18 54 89 128 169 221
	**8**	40 67 94 120 139 156 176 193	48 73 93 118 138 166 196 225	45 76 106 129 153 176 205 229	1 3 30 81 120 181 218 237	26 56 92 133 185 205 224 244	23 42 91 133 163 204 223 236
	**10**	41 59 83 103 124 141 151 167 185 198	44 64 81 100 115 134 155 175 199 223	43 67 86 103 123 141 159 176 198 227	13 26 41 66 92 119 147 190 226 246	23 54 74 99 123 145 167 190 216 240	15 26 59 95 113 130 156 183 212 238
**Test 5**	**4**	86 123 161 198	88 123 165 203	78 118 159 199	8 55 142 203	82 107 159 198	22 57 114 226
	**6**	71 90 116 154 188 218	78 103 127 157 186 211	65 101 133 162 190 222	42 106 138 203 227 253	11 41 110 152 204 248	75 133 164 194 214 237
	**8**	33 67 88 114 131 158 183 212	59 76 97 114 135 164 192 222	58 78 97 121 142 168 192 227	11 33 86 110 175 195 211 247	3 63 123 177 197 216 239 255	26 54 85 123 150 178 211 230
	**10**	57 77 100 116 132 148 166 181 201 223	36 52 70 88 109 128 146 160 183 201	64 87 101 115 128 144 161 179 196 217	32 46 62 94 121 149 171 188 209 238	8 21 50 87 104 138 148 166 192 229	9 36 88 110 123 142 163 182 216 249
**Test 6**	**4**	59 104 157 197	69 106 147 193	59 100 130 177	25 127 153 246	16 78 149 224	28 98 172 231
	**6**	54 81 108 141 175 202	55 83 115 151 177 209	41 71 104 137 163 199	36 100 122 148 178 204	29 87 116 142 163 198	13 28 69 91 137 228
	**8**	45 74 100 123 146 167 191 215	55 81 105 131 151 171 191 213	38 67 92 122 142 169 186 211	8 52 79 123 158 181 210 243	11 50 109 132 169 204 219 234	20 63 104 130 137 168 207 246
	**10**	37 53 73 90 110 131 147 170 197 222	47 69 89 105 128 143 162 179 197 219	36 61 83 99 118 137 157 175 199 216	23 42 60 77 108 132 152 182 218 235	6 33 61 108 126 158 180 207 228 245	14 71 105 126 149 173 208 219 230 244
**Test 7**	**4**	74 110 145 176	85 107 145 184	75 100 136 182	28 95 224 255	43 70 135 235	46 134 198 247
	**6**	63 84 114 143 170 201	22 76 100 132 169 221	48 79 107 130 151 191	7 30 49 115 157 194	30 71 107 144 183 242	4 55 137 166 205 251
	**8**	41 68 97 120 139 165 184 216	43 76 97 119 140 164 181 220	34 57 82 109 129 151 200 233	16 37 65 85 108 141 169 222	13 44 70 118 144 157 177 236	13 57 95 117 145 183 214 230
	**10**	53 70 89 109 122 141 159 176 195 217	25 44 66 81 103 123 142 158 180 227	55 80 105 123 133 144 156 164 184 234	14 35 57 75 88 120 141 154 173 214	11 38 58 76 100 126 149 172 189 240	17 37 74 100 124 155 175 210 227 242
**Test 8**	**4**	69 124 160 207	62 118 152 207	44 92 122 189	48 83 156 213	22 81 125 216	61 98 114 212
	**6**	50 85 114 157 184 213	54 85 116 149 176 216	38 71 103 128 192 221	6 31 73 108 214 254	49 103 122 161 190 235	14 55 109 163 195 234
	**8**	41 61 86 111 136 161 188 220	41 67 88 110 130 157 193 224	38 68 91 105 123 140 184 232	21 41 67 76 107 134 163 231	21 47 88 119 159 194 221 242	12 37 67 116 137 160 184 235
	**10**	34 47 68 88 112 132 157 180 214 236	45 70 89 108 125 137 152 164 178 206	28 42 55 76 104 132 167 194 210 230	15 38 76 102 125 160 178 209 224 243	10 33 46 73 101 138 162 204 227 242	33 47 68 93 114 132 142 153 177 213
**Test 9**	**4**	70 104 138 160	71 106 142 176	58 107 149 225	47 86 157 234	43 92 125 219	37 92 147 223
	**6**	57 82 110 133 156 177	61 92 116 137 163 187	43 74 102 126 155 206	41 93 139 167 213 221	13 79 118 156 166 205	11 71 112 155 175 239
	**8**	50 72 97 120 138 155 172 190	54 75 93 110 131 151 173 194	37 62 91 121 143 165 205 219	3 19 32 70 111 155 188 227	11 35 76 122 141 181 216 230	23 74 94 113 147 189 214 241
	**10**	49 64 79 92 105 118 137 158 173 191	50 69 90 108 121 139 155 170 187 204	38 67 85 98 113 129 145 164 196 222	7 35 69 99 115 136 157 184 210 251	5 17 37 91 113 158 202 229 247 252	25 57 90 112 141 155 174 196 216 232
**Test 10**	**4**	62 89 136 179	70 103 136 191	70 111 156 191	32 132 173 217	37 90 134 215	70 129 166 192
	**6**	52 93 129 155 187 226	54 88 116 148 184 216	50 80 111 141 171 207	51 85 123 155 187 218	14 33 61 117 187 237	41 67 108 139 182 219
	**8**	35 66 89 107 136 164 188 219	49 72 96 117 141 167 198 223	47 67 89 117 143 166 193 224	21 53 63 86 123 185 213 236	19 56 78 112 143 186 238 253	11 44 70 123 154 173 193 215
	**10**	41 67 90 114 131 153 174 200 215 232	41 62 80 96 109 128 139 157 189 227	41 61 81 102 121 136 158 172 199 232	19 37 65 91 104 134 165 194 223 246	29 50 74 98 114 137 161 190 226 240	6 28 57 105 132 157 180 199 224 239

**Table 6:** Comparison of CPU Time (in seconds) and PSNR computed by CEFO, EFO, ABC, BA, WDO, and BSA using fuzzy entropy. The bold numbers are the best values in the relevant index.

Image	K	Computational Time (CPU Time)						Peak Signal to Noise Ratio (PSNR)					
		CEFO	EFO	ABC	BA	WDO	BSA	CEFO	EFO	ABC	BA	WDO	BSA
**Test 1**	**4**	**0.17541**	0.20379	5.13759	2.45288	2.49367	2.97138	**19.0869**	18.5304	17.8020	17.3212	15.9576	16.8238
	**6**	**0.21623**	0.26617	6.63971	2.98102	3.04503	3.33423	**22.4966**	21.0851	20.8826	19.4872	19.2521	19.3995
	**8**	**0.26219**	0.30416	7.40398	3.54215	3.53203	3.96657	**23.9315**	23.3153	23.0079	23.0902	21.2553	21.9558
	**10**	**0.33284**	0.35769	8.54834	4.05983	4.12060	4.61008	**25.1603**	24.8085	24.9762	23.5623	23.5174	24.2976
**Test 2**	**4**	**0.18152**	0.19745	5.48045	2.62491	2.55021	3.05768	**18.5011**	16.6384	17.9217	14.9956	14.6621	16.8609
	**6**	**0.23014**	0.25140	6.52806	2.98864	3.11644	3.67576	21.4186	20.8410	**21.4261**	17.9962	16.1975	22.5361
	**8**	**0.26745**	0.30171	7.39266	3.54545	3.54003	4.00924	**23.4745**	23.5633	24.0574	18.3652	19.0371	22.6106
	**10**	**0.35415**	0.36493	8.32270	4.07706	4.17821	4.71338	**27.1960**	26.5362	26.5537	24.7670	19.9620	22.7476
**Test 3**	**4**	**0.19354**	0.20952	5.42962	2.54004	2.32354	3.05836	17.9266	**18.0706**	17.7559	15.0795	17.8259	16.1882
	**6**	**0.24406**	0.27724	6.51639	3.07212	3.14887	3.21041	**21.4595**	20.9724	21.0554	20.6465	20.9096	18.7586
	**8**	**0.26572**	0.30195	7.43254	3.61201	3.64706	4.05836	**23.5257**	22.7557	22.9832	22.7031	23.0729	21.4050
	**10**	**0.31104**	0.35756	8.49756	4.05537	4.27057	4.97929	**25.1107**	24.1796	24.6243	23.9076	24.8630	21.4820
**Test 4**	**4**	**0.20654**	0.22286	5.22364	2.53570	2.51094	2.89167	**18.5791**	18.5562	18.4538	17.8594	18.1050	18.4835
	**6**	**0.25613**	0.27055	6.52943	3.00267	3.05336	3.40388	**21.9962**	21.5752	21.4728	21.0931	20.4419	21.3494
	**8**	**0.29565**	0.31734	7.34861	3.53905	3.74083	4.08592	**23.6574**	22.9513	22.5091	22.4950	22.1239	21.8930
	**10**	**0.34226**	0.43022	8.48234	4.19234	4.20027	4.77867	**24.8212**	24.7081	24.3937	24.5774	23.1124	24.5733
**Test 5**	**4**	**0.20017**	0.22525	5.27645	2.53857	2.48718	2.98304	**18.0239**	17.6242	16.8089	16.3870	17.3516	15.8329
	**6**	**0.25485**	0.26024	6.89415	3.06993	3.06272	3.53148	20.3479	**20.4329**	20.0547	20.2630	19.1668	18.4271
	**8**	**0.29424**	0.32101	7.55179	3.86221	3.68722	4.09332	22.1661	21.8127	**22.1693**	21.5206	21.5001	19.8220
	**10**	**0.34480**	0.35174	8.37159	4.09532	4.24325	4.95875	**24.0138**	23.7462	23.8704	23.4936	22.2399	23.5245
**Test 6**	**4**	**0.19472**	0.20841	5.12137	2.49369	2.53905	3.08066	**18.1095**	17.6491	17.6509	17.7857	17.7293	16.8704
	**6**	**0.24263**	0.26745	6.57661	3.07078	3.06447	3.58303	**21.4831**	20.6287	21.0117	20.2254	20.2431	19.4964
	**8**	**0.30864**	0.32049	7.35641	3.57464	3.50718	4.12885	23.2290	**23.3256**	22.7773	21.8122	21.4135	20.8800
	**10**	**0.33725**	0.35708	8.54900	4.03751	4.15783	4.86694	**24.7681**	24.4737	24.5259	24.6509	23.1793	22.5603
**Test 7**	**4**	**0.20678**	0.23005	5.22556	2.53131	2.44727	3.00952	**18.8739**	18.5039	18.0277	16.4128	16.4863	16.0152
	**6**	**0.23562**	0.25809	6.62490	3.07199	3.21664	3.41591	**22.1443**	21.6213	20.0762	19.9806	20.8248	18.3119
	**8**	**0.29807**	0.30283	7.41492	3.56056	3.69153	4.05134	**23.7839**	23.1932	23.2384	22.0415	24.3675	22.0538
	**10**	**0.34217**	0.36273	8.58824	4.20910	4.36751	4.74359	**24.7433**	24.0335	24.3244	23.0629	23.8627	24.2057
**Test 8**	**4**	**0.21450**	0.22378	5.33752	2.52727	2.33784	2.93353	**18.9495**	17.4911	16.6399	16.5774	18.2614	17.6314
	**6**	**0.25068**	0.28080	6.43523	3.03905	3.08865	3.44368	21.4109	20.5791	**21.8933**	20.7661	22.1747	19.2986
	**8**	**0.31994**	0.35980	7.36667	3.57058	3.46608	4.14337	**23.0677**	22.4398	23.0619	22.3759	23.4846	21.3875
	**10**	**0.33725**	0.36888	8.48988	4.08254	4.16608	4.89443	25.4357	25.3720	23.9689	23.7873	**25.4598**	24.9694
**Test 9**	**4**	**0.19324**	0.23558	5.28317	2.55034	2.32281	3.11904	16.4568	15.9776	16.5525	16.1450	16.1994	**18.1708**
	**6**	**0.23789**	0.27384	6.58909	3.06363	3.08147	3.32353	18.4675	18.6749	**19.2639**	18.5698	18.4175	19.1084
	**8**	**0.27057**	0.29738	7.32034	3.58204	3.53299	4.04083	**21.0098**	20.5312	20.6003	20.3908	19.9597	21.0047
	**10**	**0.33151**	0.36361	8.34554	4.15394	4.26364	4.60027	22.3666	23.0655	**24.4486**	22.4630	20.8358	22.9661
**Test 10**	**4**	**0.19039**	0.23247	5.45731	2.58281	2.42582	3.10718	**18.5817**	18.4860	18.1025	17.9358	18.4517	17.3690
	**6**	**0.23656**	0.25762	6.46701	3.03982	3.12420	3.45783	**20.8938**	20.8450	20.8187	20.7592	20.5685	19.7354
	**8**	**0.26390**	0.29360	7.34870	3.59042	3.54711	3.95625	22.6241	22.3346	22.3827	22.2330	**22.6466**	22.6210
	**10**	**0.33304**	0.34301	8.49122	4.12545	4.29727	4.76364	**25.3640**	24.8916	24.8559	24.7299	23.3085	24.8469

**Table 7:** Comparison of MSSIM and FSIM computed by CEFO, EFO, ABC, BA, WDO, and BSA using fuzzy entropy. The bold numbers are the best values in the relevant index.

Image	K	Mean Structural Similarity (MSSIM)						Feature Similarity (FSIM)					
		CEFO	EFO	ABC	BA	WDO	BSA	CEFO	EFO	ABC	BA	WDO	BSA
**Test 1**	**4**	**0.97368**	0.96312	0.96192	0.94201	0.94017	0.95642	**0.75882**	0.74880	0.74221	0.72940	0.72485	0.69178
	**6**	**0.98631**	0.98280	0.98244	0.95769	0.97051	0.97305	**0.85039**	0.83938	0.81125	0.76648	0.80694	0.75111
	**8**	**0.99199**	0.98985	0.98934	0.98929	0.98299	0.98442	**0.88618**	0.87683	0.86915	0.86860	0.85435	0.83095
	**10**	**0.99361**	0.99251	0.99329	0.98959	0.98878	0.99215	**0.91098**	0.90282	0.90927	0.88384	0.89480	0.89012
**Test 2**	**4**	**0.96013**	0.95306	0.95484	0.93035	0.92588	0.95814	**0.72972**	0.68909	0.69247	0.64807	0.64540	0.65013
	**6**	**0.98459**	0.97495	0.97746	0.93722	0.94664	0.98110	**0.81315**	0.73786	0.74709	0.70236	0.67594	0.73024
	**8**	**0.99098**	0.98908	0.98343	0.96678	0.97116	0.98765	**0.86210**	0.84695	0.85625	0.71072	0.74300	0.83126
	**10**	**0.99631**	0.99389	0.99545	0.99194	0.97709	0.98899	**0.92876**	0.92497	0.91465	0.87880	0.76162	0.84512
**Test 3**	**4**	**0.97373**	0.97293	0.97077	0.90106	0.96489	0.95882	0.71919	**0.72285**	0.71887	0.68456	0.70871	0.65393
	**6**	**0.98862**	0.98623	0.98767	0.98523	0.98145	0.97832	**0.83144**	0.81185	0.82559	0.80544	0.82705	0.74121
	**8**	**0.99319**	0.99143	0.99196	0.99137	0.99274	0.98806	**0.88510**	0.87295	0.87866	0.87292	0.87457	0.84056
	**10**	**0.99513**	0.99393	0.99447	0.99282	0.99467	0.98472	**0.91471**	0.88683	0.91214	0.90164	0.90996	0.86459
**Test 4**	**4**	**0.97504**	0.97382	0.97329	0.97005	0.96999	0.97144	**0.75966**	0.75493	0.75345	0.75329	0.72873	0.73845
	**6**	**0.98817**	0.98736	0.98634	0.98489	0.98256	0.98795	**0.85825**	0.84736	0.84706	0.82680	0.80402	0.84761
	**8**	**0.99221**	0.99099	0.99161	0.99025	0.98786	0.98684	**0.89738**	0.88582	0.89347	0.88408	0.84668	0.86167
	**10**	**0.99485**	0.99452	0.99446	0.99308	0.99011	0.99385	**0.92218**	0.91879	0.91227	0.90467	0.86575	0.91742
**Test 5**	**4**	**0.97653**	0.97315	0.96886	0.96022	0.96675	0.96267	**0.76795**	0.76296	0.74913	0.75639	0.75709	0.72777
	**6**	**0.98616**	0.98513	0.98517	0.98732	0.97710	0.97627	**0.82839**	0.82738	0.81941	0.82174	0.80032	0.78086
	**8**	**0.99131**	0.98955	0.99027	0.98258	0.98718	0.98158	**0.86282**	0.85410	0.85843	0.85236	0.85559	0.82891
	**10**	**0.99470**	0.99435	0.99429	0.99253	0.98864	0.99286	**0.89313**	0.88408	0.88847	0.88520	0.88349	0.87589
**Test 6**	**4**	**0.97060**	0.96579	0.96406	0.95931	0.96633	0.95680	**0.77366**	0.74182	0.73894	0.75263	0.74499	0.71969
	**6**	**0.98591**	0.98494	0.98417	0.97846	0.98073	0.97718	**0.83568**	0.82893	0.83366	0.79984	0.81545	0.80549
	**8**	**0.99198**	0.99165	0.98932	0.98702	0.98448	0.98218	**0.88446**	0.88168	0.86619	0.85645	0.84359	0.82360
	**10**	**0.99424**	0.99330	0.99346	0.99299	0.98979	0.98715	**0.90343**	0.89737	0.90076	0.89585	0.87933	0.85478
**Test 7**	**4**	**0.97596**	0.97279	0.97349	0.95629	0.95168	0.95706	**0.75191**	0.72087	0.73196	0.69886	0.72229	0.62681
	**6**	**0.98862**	0.98840	0.98061	0.98013	0.98278	0.97028	**0.84983**	0.83905	0.79482	0.79773	0.82547	0.70824
	**8**	**0.99260**	0.99116	0.99129	0.98974	0.99020	0.98792	**0.88582**	0.87568	0.87649	0.86537	0.87662	0.83777
	**10**	**0.99407**	0.99259	0.99320	0.99084	0.99053	0.99268	**0.90659**	0.89269	0.90124	0.87211	0.88049	0.89262
**Test 8**	**4**	**0.98354**	0.97469	0.96164	0.97004	0.98276	0.97990	**0.79086**	0.76787	0.75329	0.78219	0.77483	0.78629
	**6**	**0.99111**	0.98909	0.99031	0.98855	0.99055	0.98273	**0.84716**	0.83915	0.83630	0.82678	0.83681	0.79247
	**8**	**0.99381**	0.99227	0.99103	0.99217	0.99266	0.99066	**0.86616**	0.84681	0.85892	0.84004	0.85703	0.80136
	**10**	**0.99847**	0.99600	0.99506	0.99363	0.99606	0.99554	**0.88832**	0.88298	0.88288	0.87188	0.87718	0.86633
**Test 9**	**4**	**0.97162**	0.97039	0.96942	0.96822	0.96587	0.97086	**0.81290**	0.79545	0.80453	0.78891	0.79135	0.80267
	**6**	**0.98281**	0.98209	0.98173	0.98096	0.97984	0.97906	**0.86751**	0.85710	0.85210	0.84483	0.85514	0.83386
	**8**	**0.98634**	0.98826	0.98486	0.98571	0.98601	0.98727	0.88414	0.89537	**0.90678**	0.87892	0.88468	0.87814
	**10**	**0.99255**	0.99236	0.99222	0.99119	0.98845	0.99031	**0.92326**	0.91715	0.91732	0.91380	0.89638	0.90067
**Test 10**	**4**	**0.97860**	0.97680	0.97229	0.96956	0.97424	0.97019	**0.78955**	0.78169	0.74377	0.74445	0.75473	0.78499
	**6**	**0.98606**	0.95543	0.98550	0.98345	0.98326	0.98265	0.81837	**0.82121**	0.80852	0.80624	0.79444	0.80907
	**8**	**0.99207**	0.99177	0.99129	0.98994	0.98941	0.99109	**0.86164**	0.86015	0.85335	0.84145	0.83516	0.84721
	**10**	**0.99532**	0.99500	0.99441	0.99384	0.99105	0.99423	**0.89375**	0.88711	0.87918	0.87236	0.84516	0.88534

**Table 8:** Comparison of optimal threshold values of CEFO at *K* = 4, 6, 8, and 10 using fuzzy entropy, Otsu’s and Kapur’s entropy.

Image	K	Fuzzy			Otsu			Kapur		
		R	G	B	R	G	B	R	G	B
**Test 1**	**4**	56 93 153 187	57 88 132 191	59 10 157 197	52 86 130 190	60 80 100 173	50 89 133 223	64 103 145 188	75 117 156 199	67 109 150 194
	**6**	20 59 92 128 167 199	26 47 81 114 161 203	25 48 76 111 138 195	52 74 105 161 177 217	68 80 101 124 167 190	10 55 85 105 155 212	51 84 116 150 184 212	55 87 117 147 178 212	56 87 115 150 187 221
	**8**	11 31 58 82 107 132 168 211	12 42 64 92 114 149 181 210	18 50 73 97 143 168 196 223	25 36 69 90 124 152 169 231	42 59 70 79 115 158 202 238	47 59 68 79 107 120 177 197	38 60 81 104 132 158 185 213	49 74 99 122 147 173 199 224	16 56 87 117 143 172 200 227
	**10**	14 37 55 75 90 111 130 159 182 210	11 41 61 95 122 146 168 185 208 237	14 40 64 80 105 136 161 189 212 231	45 51 67 107 119 129 165 199 222 237	41 70 72 86 91 112 156 192 210 230	35 50 82 100 101 115 139 148 178 197	12 38 58 81 106 133 161 184 203 227	45 61 82 103 124 146 168 189 210 228	17 52 74 94 116 137 163 185 212 238
**Test 2**	**4**	10 93 114 171	86 143 194 226	91 110 137 189	39 92 144 206	36 79 116 158	20 45 86 141	76 120 164 208	89 130 171 210	68 111 150 194
	**6**	18 41 75 107 147 177	87 101 133 153 192 234	54 72 97 121 156 192	22 59 88 121 183 197	29 61 90 129 165 179	18 23 36 64 95 233	20 58 96 135 173 213	19 58 99 138 177 215	41 74 106 137 165 199
	**8**	15 27 44 59 96 127 171 204	14 22 43 65 117 144 190 222	39 61 82 117 148 176 195 223	26 47 103 127 151 181 222 235	5 27 61 84 102 120 132 170	13 45 55 61 104 140 180 186	16 48 79 109 140 170 200 228	17 52 84 114 147 175 201 228	15 41 67 92 117 143 169 200
	**10**	32 55 75 108 144 171 195 215 226 239	5 50 65 85 102 121 139 167 197 228	31 47 64 87 108 125 158 179 209 239	18 23 47 56 99 115 143 184 194 237	22 71 76 78 99 108 138 158 196 212	18 43 56 97 129 145 162 234 235 237	13 35 56 81 104 130 155 179 205 230	15 40 65 92 112 134 159 183 206 229	13 37 58 81 102 123 143 163 190 209
**Test 3**	**4**	44 92 139 188	31 92 139 188	27 97 138 185	63 113 115 170	93 99 136 192	90 113 150 192	49 98 145 202	25 85 133 192	58 122 157 202
	**6**	32 62 93 128 173 199	19 53 90 119 162 206	36 71 105 136 183 208	48 78 103 126 153 190	36 71 114 168 185 203	18 30 101 133 159 201	30 70 102 127 166 200	20 65 99 143 181 208	26 80 104 135 168 202
	**8**	22 43 68 91 112 138 172 208	17 51 73 98 123 148 181 207	11 36 58 88 106 141 170 213	15 68 93 126 130 160 177 225	7 40 95 123 146 172 186 209	18 80 114 137 167 197 206 239	13 33 60 87 125 157 185 214	29 52 73 97 124 161 187 222	12 42 61 86 108 137 167 205
	**10**	26 39 55 69 86 110 131 154 175 207	42 29 56 79 104 121 141 163 197 219	9 25 49 82 109 132 154 176 199 222	1 37 64 71 99 122 151 176 212 231	13 51 74 75 104 137 170 197 215 246	51 82 106 111 130 168 181 203 233 241	11 32 51 73 100 132 154 173 199 223	14 37 55 75 93 109 123 149 181 212	16 44 66 99 130 150 166 187 208 225
**Test 4**	**4**	52 84 127 164	67 102 141 204	49 74 111 155	36 86 170 196	66 107 139 229	41 83 91 146	55 94 133 173	68 124 178 218	40 79 126 203
	**6**	38 62 97 137 172 195	48 71 111 144 175 213	43 77 109 147 178 235	33 63 93 127 163 215	36 66 112 138 168 231	22 48 83 135 183 206	51 86 121 155 190 224	46 80 116 149 183 219	27 55 83 118 155 203
	**8**	13 31 54 92 110 142 175 198	35 60 86 114 137 169 200 226	24 41 64 94 129 156 193 224	33 49 87 98 142 193 231 251	6 25 60 85 118 156 173 218	19 37 39 41 47 67 103 150	34 58 87 116 145 173 201 231	35 61 86 112 139 165 192 223	26 54 79 106 129 155 182 204
	**10**	6 25 43 64 84 106 132 152 172 189	7 27 39 52 75 99 130 157 190 221	15 25 48 78 103 130 157 178 219 240	39 48 73 105 135 152 156 167 197 225	26 39 48 70 106 136 158 191 199 220	9 28 49 66 71 94 131 159 170 186	32 56 78 99 123 145 168 190 211 235	26 48 69 90 112 133 157 180 204 229	21 43 63 80 101 121 145 165 183 204
**Test 5**	**4**	52 98 149 198	55 89 164 215	77 125 162 209	95 147 188 198	59 135 142 212	81 116 164 203	74 110 145 194	78 120 160 205	22 88 147 218
	**6**	22 63 93 131 179 225	16 69 97 125 158 204	15 61 97 131 166 207	92 132 165 192 233 243	78 116 119 138 187 229	68 125 134 158 190 248	66 92 118 145 180 216	67 98 126 160 191 223	22 62 99 142 180 219
	**8**	16 59 77 94 116 134 162 199	20 58 75 98 129 155 185 214	19 56 77 115 145 173 196 227	89 111 120 148 188 207 240 246	62 96 152 160 186 194 199 215	17 76 100 131 141 169 197 231	65 90 115 140 162 184 204 225	64 88 112 135 158 182 206 227	18 59 89 119 146 172 197 221
	**10**	13 29 57 77 93 107 130 165 188 214	14 30 62 78 94 126 152 179 205 231	19 44 56 72 89 109 129 159 176 205	36 66 73 82 104 120 126 147 181 210	60 104 127 170 175 181 185 190 206 221	35 58 87 97 135 174 175 189 214 222	54 71 87 106 127 145 168 189 208 225	62 81 100 120 143 163 183 201 218 233	22 52 81 108 133 154 176 197 217 235
**Test 6**	**4**	55 97 158 195	64 99 153 190	46 75 131 179	54 104 163 222	69 112 141 214	37 76 121 191	60 109 155 201	66 112 152 199	58 110 156 213
	**6**	43 60 91 118 165 202	25 38 76 119 165 205	47 69 97 133 155 190	49 60 78 135 146 239	52 64 81 132 190 233	28 57 96 130 201 227	45 79 112 147 181 214	54 91 126 155 185 217	38 78 119 155 188 222
	**8**	16 42 73 91 129 157 190 221	10 34 52 79 108 136 168 207	23 39 64 86 120 137 172 202	45 50 81 113 133 174 184 197	20 37 51 76 112 141 168 205	3 11 39 75 110 120 187 211	35 60 86 112 139 166 194 222	44 68 91 116 138 163 189 217	32 58 86 112 138 164 196 226
	**10**	10 32 50 77 99 134 162 192 209 228	13 36 47 69 107 140 164 185 200 223	14 33 60 82 101 130 156 183 208 228	41 49 70 99 111 112 171 191 237 245	42 60 61 85 102 148 186 248 249	13 24 50 87 104 131 137 156 178 229	11 36 59 82 109 135 161 187 209 232	38 58 79 100 119 140 165 189 208 228	15 37 60 86 110 133 158 186 211 232
**Test 7**	**4**	66 95 139 176	25 66 108 157	23 88 134 220	85 132 159 183	66 104 171 190	73 104 155 175	75 115 155 193	81 119 157 195	47 88 131 180
	**6**	17 33 80 120 168 205	20 80 108 133 164 189	13 59 84 102 128 151	77 106 144 188 212 224	51 65 76 110 152 200	45 49 79 90 131 154	63 96 128 160 194 225	47 77 106 134 163 195	17 47 79 112 144 180
	**8**	20 54 79 106 126 146 169 209	13 50 80 102 132 166 197 223	19 40 63 88 119 148 200 228	48 67 83 123 141 182 224 234	79 100 140 150 165 183 214 254	6 45 49 80 107 131 165 205	38 61 84 110 144 169 195 223	60 88 116 142 168 196 216 237	12 31 56 82 111 133 154 180
	**10**	10 30 41 63 89 110 129 154 178 216	14 54 68 82 106 132 169 197 220 243	15 41 66 85 105 133 151 190 214 233	38 63 76 101 128 131 157 164 185 186	27 41 83 91 103 119 157 184 219 226	14 55 72 83 86 115 124 157 164 214	41 61 82 101 120 142 167 194 216 237	46 69 92 114 138 158 176 196 216 239	17 42 67 84 111 130 144 161 179 196
**Test 8**	**4**	48 73 173 212	62 107 141 207	43 79 124 215	115 174 190 225	92 120 136 156	46 100 139 150	59 116 149 202	83 122 182 218	55 96 130 164
	**6**	38 64 101 141 181 224	32 61 85 126 155 196	38 61 91 126 197 218	54 146 170 197 219 232	98 119 143 156 167 212	12 42 50 121 142 182	42 81 118 148 179 209	45 83 116 147 182 218	25 49 72 101 131 164
	**8**	15 41 64 85 110 133 175 213	44 34 74 110 135 158 179 203	25 40 69 104 136 182 207 231	65 72 140 154 178 188 218 239	69 111 141 150 170 218 245	28 29 72 99 116 128 188 200	37 61 90 120 144 171 200 226	46 73 101 126 152 182 204 225	20 43 61 80 99 121 143 164
	**10**	18 39 58 87 115 137 172 186 210 230	21 36 63 79 111 129 150 180 208 227	28 47 74 90 116 131 153 178 208 226	28 57 59 113 123 163 168 191 213 214	39 70 72 102 128 154 169 183 191 196	9 21 27 77 79 128 168 185 187 197	35 58 80 100 121 142 165 187 207 230	36 47 71 91 116 139 164 183 214 228	20 37 53 70 89 107 126 143 164 182
**Test 9**	**4**	47 74 107 149	55 90 132 167	42 66 117 149	40 93 131 211	80 137 195 205	29 81 119 172	76 123 167 212	77 129 180 227	43 90 140 194
	**6**	13 43 62 92 126 165	15 50 89 125 160 195	19 45 81 115 151 230	57 73 109 164 194 230	64 105 136 166 201 231	15 61 91 114 123 165	62 97 132 167 203 236	62 95 128 162 195 231	31 63 95 126 158 194
	**8**	14 36 53 75 92 123 152 173	11 40 55 77 101 126 151 183	26 41 66 91 128 155 200 233	45 62 101 143 153 191 200 229	45 92 128 151 156 193 199 217	12 65 78 106 118 135 169 214	55 83 110 138 162 188 213 238	53 80 108 137 164 191 215 238	25 47 70 97 119 142 171 199
	**10**	13 23 41 59 82 106 126 158 181 198	11 43 54 72 89 105 134 163 188 213	24 47 71 94 118 140 160 177 208 237	30 48 87 101 131 159 164 220 227 253	30 50 59 64 107 112 136 166 189 228	12 20 47 80 93 106 122 132 150 165	47 68 92 115 140 160 182 201 219 239	38 55 74 95 118 142 164 187 212 238	19 36 54 72 91 111 133 156 176 194
**Test 10**	**4**	48 76 132 175	31 69 117 169	53 86 116 200	57 112 164 206	63 108 179 202	47 95 172 216	49 91 133 182	63 111 157 206	59 99 148 192
	**6**	34 57 90 127 166 196	53 90 116 142 180 214	42 56 104 136 169 220	18 53 72 129 133 155	55 85 150 177 181 231	38 75 127 175 203 229	30 67 106 136 177 217	46 79 111 141 170 2070	44 78 114 152 190 225
	**8**	19 33 62 91 133 165 195 214	20 26 57 90 112 150 195 220	12 38 55 88 113 150 186 229	34 71 116 118 139 141 159 199	15 42 60 103 149 155 170 210	6 45 70 127 139 200 219 248	22 52 79 109 133 161 189 219	39 67 95 124 153 180 206 230	40 66 91 116 142 167 194 224
	**10**	16 35 54 71 93 119 141 169 196 231	9 26 36 51 72 106 132 166 195 224	10 26 41 61 77 108 134 160 185 213	13 28 49 58 95 105 127 145 151 184	26 65 103 145 169 178 183 193 207 239	19 49 63 107 161 187 191 226 242 249	21 42 67 95 121 148 173 197 217 232	34 55 78 101 122 166 186 207 231	29 47 70 92 117 139 161 183 204 226

**Table 9:** Comparison of CPU Time, PSNR, MSSIM, and FSIM computed by CEFO at *K* = 4, 6, 8, 10 using fuzzy entropy, Otsu’s and Kapur’s. The bold numbers are the best values in the relevant index.

Image	K	CPU Time			PSNR			MSSIM			FSIM		
		Fuzzy	Otsu’s	Kapur	Fuzzy	Otsu’s	Kapur	Fuzzy	Otsu’s	Kapur	Fuzzy	Otsu’s	Kapur
**Test 1**	**4**	0.17541	**0.11919**	0.18065	**19.0869**	16.0378	16.0378	**0.97368**	0.95866	0.94199	**0.75882**	0.75699	0.73718
	**6**	0.21623	**0.12506**	0.24117	**22.4966**	19.0529	19.0529	**0.98631**	0.96735	0.97092	**0.85039**	0.79878	0.81115
	**8**	0.26219	**0.13443**	0.25866	**23.9315**	21.3692	21.9768	**0.99199**	0.98135	0.98558	**0.88618**	0.83430	0.86369
	**10**	0.33284	**0.15629**	0.27287	**25.1603**	22.8071	23.8019	**0.99361**	0.98815	0.99060	**0.91098**	0.85722	0.89851
**Test 2**	**4**	0.18152	**0.10603**	0.24967	**18.5011**	18.4665	18.4854	**0.96013**	0.95859	0.95939	**0.72972**	0.72349	0.71672
	**6**	0.23014	**0.11012**	0.25587	**21.4186**	20.9856	21.2795	**0.98459**	0.98013	0.98049	**0.81315**	0.80687	0.80330
	**8**	0.26745	**0.12666**	0.26542	**23.4745**	23.2473	23.2436	**0.99098**	0.98926	0.98958	**0.86210**	0.85725	0.86029
	**10**	0.35415	**0.14591**	0.30354	**27.1960**	24.6605	26.8256	**0.99631**	0.99286	0.99584	**0.92876**	0.87911	0.92489
**Test 3**	**4**	0.19354	**0.11462**	0.39732	**17.9266**	17.5340	17.7798	**0.97373**	0.96767	0.97158	**0.71919**	0.70489	0.71365
	**6**	0.24406	**0.12074**	0.45047	**21.4595**	20.4054	21.3185	**0.98862**	0.98374	0.98793	**0.83144**	0.79214	0.82536
	**8**	0.26572	**0.13348**	0.48285	**23.5257**	21.5710	22.7384	**0.99319**	0.98726	0.99194	**0.88510**	0.82899	0.87669
	**10**	0.31104	**0.16254**	0.21154	**25.1107**	23.3835	24.3602	**0.99513**	0.99227	0.99417	0.91471	0.88218	**0.92049**
**Test 4**	**4**	0.20654	**0.09202**	0.21866	**18.5791**	18.4569	18.4989	**0.97504**	0.94314	0.97497	**0.75966**	0.74368	0.74512
	**6**	0.25613	**0.12600**	0.22851	21.9962	21.7162	22.0191	**0.98817**	0.98612	0.98724	0.85825	0.84935	**0.85897**
	**8**	0.29565	**0.15338**	0.24107	**23.6574**	23.0068	23.5891	0.99221	0.99073	**0.99239**	**0.89738**	0.88483	0.89628
	**10**	0.34226	**0.16649**	0.27016	**24.8212**	24.5519	24.7934	**0.99485**	0.99308	0.99460	**0.92218**	0.91235	0.92176
**Test 5**	**4**	0.20017	**0.10569**	0.23254	**18.0239**	16.4797	17.2779	**0.97653**	0.96171	0.97062	**0.76795**	0.74241	0.76655
	**6**	0.23485	**0.12750**	0.24186	**20.3479**	17.9620	20.0647	**0.98616**	0.97050	0.98300	**0.82839**	0.81379	0.82815
	**8**	0.29424	**0.15211**	0.27258	**22.1661**	19.6993	21.4806	**0.99131**	0.97874	0.98620	0.86282	0.83396	**0.87859**
	**10**	0.34480	**0.16173**	0.29775	**24.0138**	21.9181	23.1757	**0.99470**	0.98889	0.99059	**0.89313**	0.85475	0.89169
**Test 6**	**4**	0.19472	**0.10286**	0.24964	**18.1095**	17.8512	17.9759	**0.97060**	0.97041	0.96622	**0.77366**	0.76319	0.74541
	**6**	0.24263	**0.12325**	0.28237	**21.4831**	20.4060	20.6824	**0.98591**	0.98186	0.98165	**0.83568**	0.81530	0.82596
	**8**	0.30864	**0.15339**	0.29413	**23.2290**	22.1354	22.9859	**0.99198**	0.98855	0.98949	**0.88446**	0.85408	0.87426
	**10**	0.33725	**0.18392**	0.32098	24.7681	22.8894	**25.0296**	0.99424	0.98975	**0.99350**	**0.90343**	0.86898	0.90198
**Test 7**	**4**	0.20678	**0.10402**	0.24912	**18.8739**	17.8704	17.8217	**0.97596**	0.96464	0.96471	**0.75191**	0.72297	0.74535
	**6**	0.23562	**0.10956**	0.25531	**22.1443**	20.3251	21.6210	**0.98862**	0.97872	0.98609	**0.84983**	0.79339	0.84398
	**8**	0.29807	**0.12937**	0.27367	**23.7839**	20.7719	23.1949	**0.99260**	0.97916	0.99048	**0.88582**	0.81106	0.87668
	**10**	0.34217	**0.14226**	0.27555	**24.7433**	23.1843	24.3686	**0.99407**	0.99097	0.99363	0.90659	0.85053	**0.90773**
**Test 8**	**4**	0.21450	**0.10617**	0.23422	18.9495	**19.0541**	18.9415	0.98354	0.98371	**0.98401**	0.79086	0.79082	**0.79759**
	**6**	0.25068	**0.12896**	0.26131	21.4109	21.1411	**21.4439**	**0.99111**	0.98687	0.99032	**0.84716**	0.80299	0.81085
	**8**	0.31994	**0.14136**	0.28517	23.0677	23.2842	**23.5914**	0.99381	0.99375	**0.99576**	0.86616	0.85771	**0.87685**
	**10**	0.33725	**0.18727**	0.29926	**25.4357**	22.6056	25.4062	**0.99847**	0.99165	0.99681	0.88832	0.82970	**0.89139**
**Test 9**	**4**	0.19324	**0.11092**	0.23595	16.4568	16.3593	**17.1580**	**0.97162**	0.97064	0.96985	**0.81290**	0.80596	0.81157
	**6**	0.23789	**0.12999**	0.17958	18.4675	18.0109	**19.0843**	0.98281	0.98205	**0.98394**	0.86751	0.86452	**0.89018**
	**8**	0.27057	**0.14229**	0.22847	21.0098	21.0045	**21.3567**	0.98634	0.98629	**0.98699**	0.88414	0.87084	**0.90497**
	**10**	0.33151	**0.18128**	0.25452	22.3666	22.3547	**23.1507**	0.99255	0.99251	**0.99428**	0.92326	0.91262	**0.93015**
**Test 10**	**4**	0.19039	**0.10466**	0.20511	**18.5817**	17.9607	18.0765	0.97860	0.97268	**0.97934**	**0.78169**	0.76586	0.77935
	**6**	0.23656	**0.11261**	0.24046	20.8938	20.8925	**21.0027**	**0.98606**	0.98526	0.98586	0.82121	0.82907	**0.83085**
	**8**	0.26390	**0.12200**	0.24834	22.6241	21.6691	**23.1804**	**0.99207**	0.98882	0.99119	**0.86015**	0.83365	0.85931
	**10**	0.33304	**0.12677**	0.27179	**25.3640**	23.8567	25.0886	**0.99532**	0.99289	0.99428	**0.88711**	0.88195	0.88555

**Table 10:** Comparison of *p*-values between CEFO and other algorithms based on Fuzzy entropy.

Dependent Variable	Proposed Algorithm	Algorithms	*p*-Value	Dependent Variable	Proposed Algorithm	Algorithms	*p*-Value
**CPU Time**	CEFO	EFO	0.038791(*)	**MSSIM**	CEFO	EFO	0.201224
		ABC	3.76E-50(*)			ABC	0.157924
		BA	1.08E-46(*)			BA	0.006606(*)
		WDO	1.23E-43(*)			WDO	0.004560(*)
		BSA	1.95E-47(*)			BSA	0.006092(*)
**PSNR**	CEFO	EFO	0.457477	**FSIM**	CEFO	EFO	0.364938
		ABC	0.466803			ABC	0.297925
		BA	0.037665(*)			BA	0.034212(*)
		WDO	0.021986(*)			WDO	0.016480(*)
		BSA	0.020720(*)			BSA	0.003728(*)
